# The Diagnostic Accuracy of Syndromic Management for Genital Ulcer Disease: A Systematic Review and Meta-Analysis

**DOI:** 10.3389/fmed.2021.806605

**Published:** 2022-01-03

**Authors:** Andre J. W. Loh, Ee Lynn Ting, Teodora E. Wi, Philippe Mayaud, Eric P. F. Chow, Nancy Santesso, Jane Falconer, Richard Ofori-Asenso, Jason J. Ong

**Affiliations:** ^1^Central Clinical School, Monash University, Melbourne, VIC, Australia; ^2^Global HIV, Hepatitis and STI Programmes, World Health Organization, Geneva, Switzerland; ^3^Faculty of Infectious and Tropical Diseases, London School of Hygiene and Tropical Medicine, London, United Kingdom; ^4^Melbourne Sexual Health Centre, Alfred Health, Carlton, VIC, Australia; ^5^Centre for Epidemiology and Biostatistics, Melbourne School of Population and Global Health, The University of Melbourne, Melbourne, VIC, Australia; ^6^Department of Health Research Methods, Evidence, and Impact, McMaster University, Hamilton, ON, Canada; ^7^Library & Archives Service, London School of Hygiene & Tropical Medicine, London, United Kingdom; ^8^Monash Outcomes Research and Health Economics, Department of Epidemiology and Preventive Medicine, Monash University, Melbourne, VIC, Australia; ^9^Real World Data Enabling Platform, Roche Products Ltd., Welwyn Garden City, United Kingdom

**Keywords:** genital ulcer disease, herpes, syphilis, syndromic algorithm, systematic review and meta-analysis, chancroid

## Abstract

**Objectives:** Genital Ulcer Disease (GUD) carries a significant disease burden globally. With limited access to diagnostics, the 2001 World Health Organization (WHO) sexually transmitted illnesses (STI) guidelines proposed a syndromic management algorithm that required a clinical decision to determine the management of GUD. We assessed the diagnostic accuracy of this algorithm.

**Methods:** We conducted a systematic review (Prospero: CRD42020153294) using eight databases for publications between 1995 and January 2021 that reported primary data on the diagnostic accuracy of clinical diagnosis to identify aetiological agents of GUD. Titles and abstracts were independently assessed for eligibility, and data were extracted from full texts for sensitivity/specificity. A hierarchical logistic regression model was used to derive pooled sensitivity and specificity. We used GRADE to evaluate the certainty of evidence.

**Results:** Of 24,857 articles, 151 full texts were examined and 29 included in the analysis. The majority were from middle-income countries [(14/29 (48%) lower middle, 10/29 (34%) upper middle)]. We pooled studies where molecular testing was using to confirm the aetiology of GUD: 9 studies (12 estimates) for herpes, 4 studies (7 estimates) for syphilis, and 7 studies (10 estimates) for chancroid. The pooled sensitivity and specificity of GUD for the detection of herpes was 43.5% [95% confidence interval (CI): 26.2–62.4], and 88.0% (95% CI: 67.0–96.3), respectively (high certainty evidence); and for syphilis were 52.8% (95% CI: 23.0–80.7), and 72.1% (95% CI: 28.0–94.5) (moderate certainty evidence); and for chancroid were 71.9% (95% CI: 45.9–88.5) and 53.1% (95% CI: 36.6–68.9) (moderate certainty evidence), respectively.

**Conclusion:** Algorithms requiring a clinical diagnosis to determine and treat the aetiology of GUD have poor sensitivities for syphilis and herpes simplex virus, resulting in significant numbers of missed cases. There is an urgent need to improve access to affordable and efficient diagnostics (e.g., point-of-care tests) to be incorporated into GUD algorithms to better guide appropriate management.

**Systematic Review Registration:** PROSPERO, identifier: CRD42020153294.

## Introduction

Sexually transmitted infections (STIs) are a major global public health problem, with significant economic, health, and social impact. In 2016, an estimated 376 million new STI cases were diagnosed (1). To curb the rising pandemic of STIs, the World Health Organization (WHO) proposed adoption of the Global Health Sector Strategy on STIs in 2016 to rapidly scale up evidence-based interventions and services to end STIs as a public health concern by 2030 (2). One area of focus was tackling the incredibly high incidence of STIs in developing countries. Developing countries bear the largest burden of disease, where STIs can account for 17% of economic losses caused by ill-health (3).

There is often a lack of adequate and affordable laboratory infrastructure for accurate aetiological diagnosis of STIs in these resource-limited countries. Guidelines for managing STIs based on a syndromic algorithm were first promoted by the WHO in 1991 and updated in 2001 to address this barrier (4). The syndromic management of STIs refers to identifying the presence of a distinct group of symptoms, including genital ulcers, urethral discharge, anorectal disease and lower abdominal pain, and recognising them to be part of an “STI syndrome.” Clinicians will treat these syndromes empirically, covering the common or most serious organisms for that particular syndrome. Eighty-three countries have adapted their national STI management guidelines to a similar approach proposed by the WHO STI clinical guidelines, suggesting its popularity (5).

Of the STI syndromes, genital ulcer disease (GUD) carries one of the most significant disease burdens. In 2016 alone, 187 million people globally, or 5% of the world's population, had at least one episode of herpes simplex virus (HSV)-related GUD (6). On top of its high global incidence, GUD can have profound and long-lasting health impacts. Women acquiring primary HSV in the third trimester of pregnancy may result in congenital herpes, leading to neurocognitive problems, developmental delays, or infant death. Syphilis, another cause of GUD, can equally cause profound and long-lasting health impacts, especially for pregnant women and infants. Untreated pregnant women with syphilis have up to 70% chance of transmitting the disease to their foetus, and 40% of these pregnancies result in perinatal death (7). There is also an association between ulcerative STIs and the acquisition of HIV (8). While we have molecular-based tests that allow quick and convenient aetiological diagnosis of GUD to guide appropriate treatment in high-income countries, they are often not widely available in low- to middle-income countries. When available, point of care testing (POCT) is preferable as obtaining a diagnosis and targeted treatment on the same day can prevent further transmission during the time required before a result is available to tailor treatment of infected individuals (9).

While the 2001 WHO STI management guidelines for all STI syndromes was originally established with a “syndromic management” approach, the specific algorithm targeting GUD includes elements of both a syndromic approach (determined solely by the presence of an ulcer) and clinical diagnosis (clinical evaluation to determine the likely aetiology of the ulcer) before appropriate treatment is administered. The presence of vesicles or small ulcers with a history of recurrent vesicles would indicate necessary treatment for HSV, while the absence of the above would be an indication to treat for syphilis and chancroid.

A previous review published in 2000 evaluated the diagnostic accuracy of STI syndromic management (including five studies for GUD) (10). Since then, the underlying aetiology of GUD has changed [e.g., disappearance of chancroid as an important cause of GUD for many parts of the world (11) and the rise of HSV as the dominant aetiology (12)], and more accurate diagnostics using molecular assays are now available. Therefore, this systematic review aimed to update the evidence for the diagnostic accuracy of the currently used 2001 WHO guideline for the management of GUD. These findings were used to update the WHO guidelines for the management of symptomatic STIs (13).

## Methods

The systematic review was conducted with the guidance of the Cochrane Handbook 5.1 (14). Eight databases (OvidSP Embase, OvidSP Global Heath, OvidSP Northern Light Life Sciences Conference Abstracts, Ebsco CINAHL Plus, Ebsco Africa-Wide Information, Clarivate Analytics Web of Science Core Collection, and BIREME/PAHO/WHO Virtual Health Library LILACS) were searched for papers published from 1995 until 11th January 2021. The systematic review was registered in PROSPERO (CRD42020153294).

### Study Eligibility Criteria

We included studies with primary data (1) comparing clinical diagnosis of aetiological causes of GUD against laboratory-confirmed causes of GUD and (2) diagnostic accuracy of the GUD syndromic approach in detecting STIs. We included randomised controlled trials and observational studies. Studies were excluded if they contained no original data, included duplicated results from another study, were solely evaluating the diagnostic accuracy of laboratory aetiological diagnosis methods of GUD without comparison to clinical diagnosis methods or were studies restricting the study population to STI syndromes other than GUD.

### Search Method and Data Extraction

The search was constructed using three concepts: (1) syndromic management; (2) genital ulcer disease; and (3) diagnostic accuracy. More details of the search strategy are presented in the Appendix. All citations identified by our searches were imported into EndNote X9 software. Once duplicates were identified and removed, two independent reviewers (ET, AL) screened the titles and abstracts, then the full-text of potentially relevant papers; discrepancies in screening were resolved by a third reviewer (JO). Relevant data were extracted from deduplicated full publications.

### Statistical Analysis

To ensure consistency of a reference diagnostic, we pooled studies if they used a molecular assay to confirm the aetiology of GUD. We used hierarchical (multilevel) models using binomial data structures, i.e., hierarchical logistic regression model using STATA version 16 (StataCorp. 2019. *Stata Statistical Software: Release 16*. College Station, TX: StataCorp LLC). We reported the pooled sensitivity, specificity, positive and negative likelihood ratios, and diagnostic odds ratio. The positive likelihood ratio expresses how many times more likely people with the condition receive a positive test result than those who do not have the condition. In contrast, the negative likelihood ratio expresses how likely it is that people with the condition will receive a negative test result than those who do not have the condition. The inverse of the negative likelihood ratio (1/LR–) can be compared with the positive likelihood ratio to indicate whether the positive or negative test result has a greater impact on the odds of disease.

We also present the summary receiver operating characteristic (SROC) curve from the hierarchical summary receiver operating characteristic (HROC) model (15), the prediction region (i.e., for the forecast of the true sensitivity and specificity in a future study). Plotting the summary operating point and its confidence region allowed us to graphically display the trade-off between sensitivity and specificity. Forest plots were used to show within-study estimates and confidence intervals for sensitivity and specificity separately. We only included papers where true positives, false positives, true negatives, and false negatives could be calculated in the meta-analyses. Heterogeneity across estimates was assessed using the *I*^2^ statistic, and publication bias was evaluated using Deek's asymmetry test. To explain the effects of heterogeneity, univariable and multivariable meta-regression was conducted using the following variables: country income level, year of publication and recruitment site. Included studies were evaluated using the QUADAS-2 checklist (rather than Johanna Briggs Institute checklists as per PROSPERO research plan) as QUADAS-2 is the recommended checklist for diagnostics when using the GRADE approach (16). We assessed the certainty of the evidence using the GRADE (17, 18). We report our review following the preferred reporting items for systematic reviews and meta-analyses (PRISMA) guidelines (19).

Given that the positive and negative predictive value will depend on a pathogen's background prevalence, we present Supplementary Tables to show the effect of the pooled sensitivity and specificity on the number of missed and overtreated cases over a range of background prevalence.

### Role of the Funding Source

The WHO funded the study and helped with the study design, analysis, interpretation of data, writing of the report, and the decision to submit the paper for publication.

## Results

**Figure 1:**
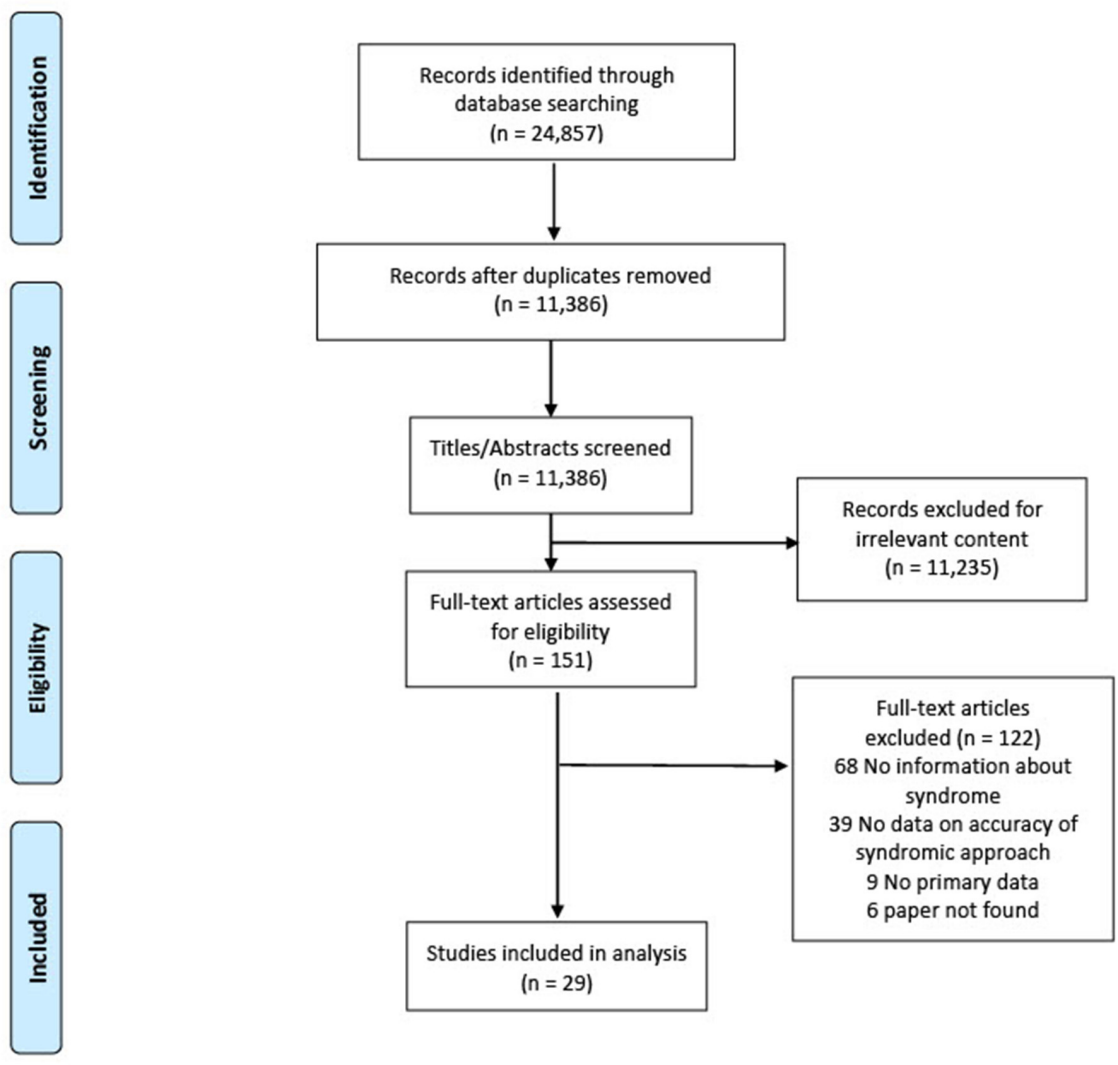
PRISMA flowchart.

We found most countries adapted the 2001 WHO algorithm for GUD into their local contexts rather than following it strictly. In the absence of data that specifically evaluated the WHO algorithm, we included studies reporting the association between the accuracy of a clinical diagnosis (e.g., clinicians may use the presence of vesicular lesions as more suggestive of HSV) compared with laboratory-based STI diagnosis. Figure 1 presents the PRISMA flowchart. Of 24,857 articles, 151 full texts were examined, and 29 included in the analysis: 17 studies examined the accuracy of a clinical diagnosis, and 12 studies examined the accuracy of the presence of GUD compared with laboratory-based STI diagnosis. Table 1 summarises the characteristics of included studies. Most studies were published before 2010, from middle-income countries, and patients were recruited primarily from sexual health clinics.

**Table 1:** Summary of included studies.

**Year of study**	***n*** **(%)**
<2000	14 (48)
2001–2010	10 (34)
>2010	5 (17)
**Country income level**	
Low	2 (7)
Low-middle	14 (48)
Upper-middle	10 (34)
High	3 (10)
**Recruitment site**	
Sexual health clinic	19 (66)
Hospital	4 (14)
Community	3 (10)
General practise	3 (10)

### Diagnostic Accuracy of Clinical Diagnosis of GUD for Detection of STIs

#### Any STI

Only one study provided two estimates (Supplementary Table 1).

#### Herpes

**Figure 2:**
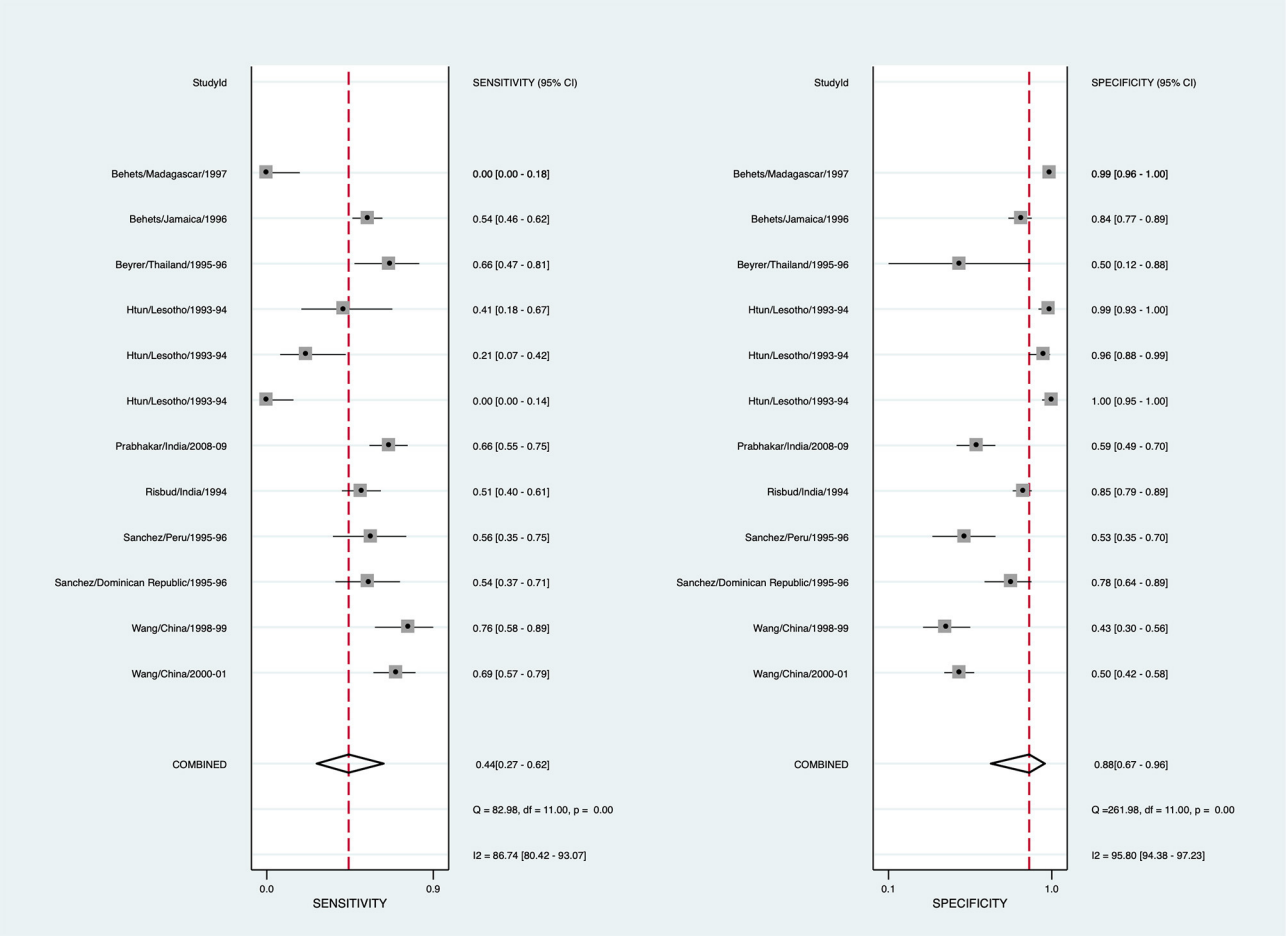
Forest plot of the sensitivity and specificity of clinical diagnosis to detect herpes simplex virus in genital ulcer disease.

To arrive at a presumptive diagnosis of herpes based on clinical evaluation among individuals with GUD, 15/29 studies provided 20 estimates, and 9 studies provided 12 estimates for the meta-analysis (Supplementary Table 2). The pooled sensitivity for detecting herpes through clinical diagnosis was 43.5% (95% CI: 26.2-62.4, *I*^2^ = 86.7), and pooled specificity was 88.0% (95% CI: 67.0–96.3, *I*^2^ = 95.8) (Figure 2). The diagnostic odds ratio was 5.63 (95% CI: 3.04–10.43). The positive likelihood ratio was 3.62 (95% CI: 1.74–7.54), and the negative likelihood ratio was 0.64 (95% CI: 0.53–0.78). The inverse negative likelihood ratio was 1.56 (95% CI: 1.28–1.90). The number of missed and overtreated cases over different background prevalence is in Supplementary Table 3. The summary receiver operating curve is in Supplementary Figure 1.

#### Syphilis

**Figure 3:**
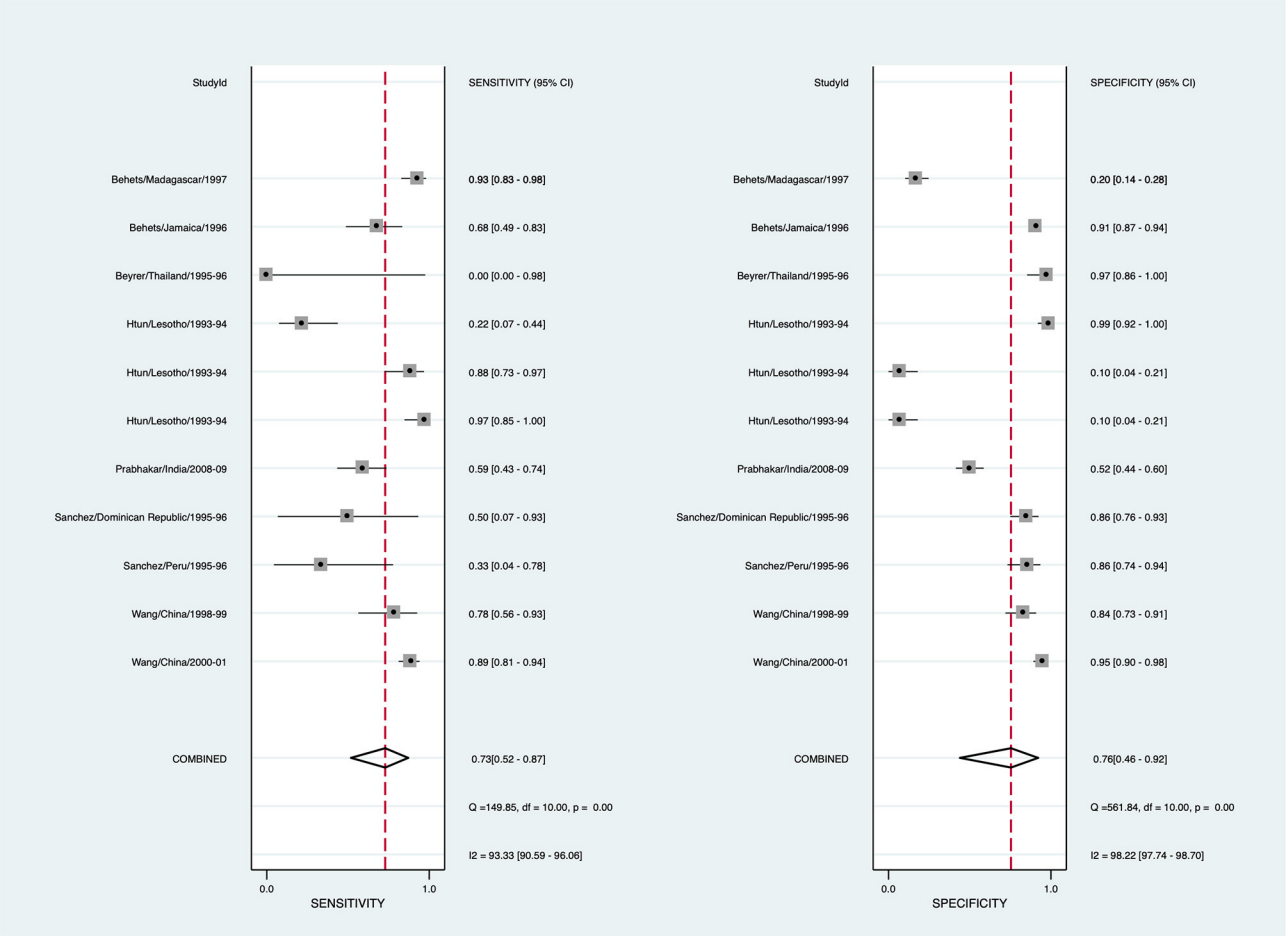
Forest plot of the sensitivity and specificity of clinical diagnosis to detect syphilis in genital ulcer disease.

To arrive at a presumptive diagnosis of syphilis based on clinical evaluation, 15/29 studies provided 22 estimates, and 8 studies provided 11 estimates for the meta-analysis (Supplementary Table 4). The pooled sensitivity for detecting syphilis through clinical diagnosis was 72.8% (95% CI: 51.4–87.1, *I*^2^ = 93.3), with the pooled specificity being 76.4% (95% CI: 45.4–92.6, *I*^2^ = 98.7) (Figure 3). The diagnostic odds ratio is 8.65 (95% CI: 3.20–23.38). The positive likelihood ratio is 3.08 (95% CI: 1.27–7.46), and negative likelihood ratio is 0.36 (95% CI: 0.21–0.60). The inverse of the negative likelihood ratio is 2.81 (95% CI: 1.68–4.69). The number of missed and overtreated cases over different background prevalence is in Supplementary Table 5. The summary receiver operating curve is in Supplementary Figure 2.

#### Chancroid

**Figure 4:**
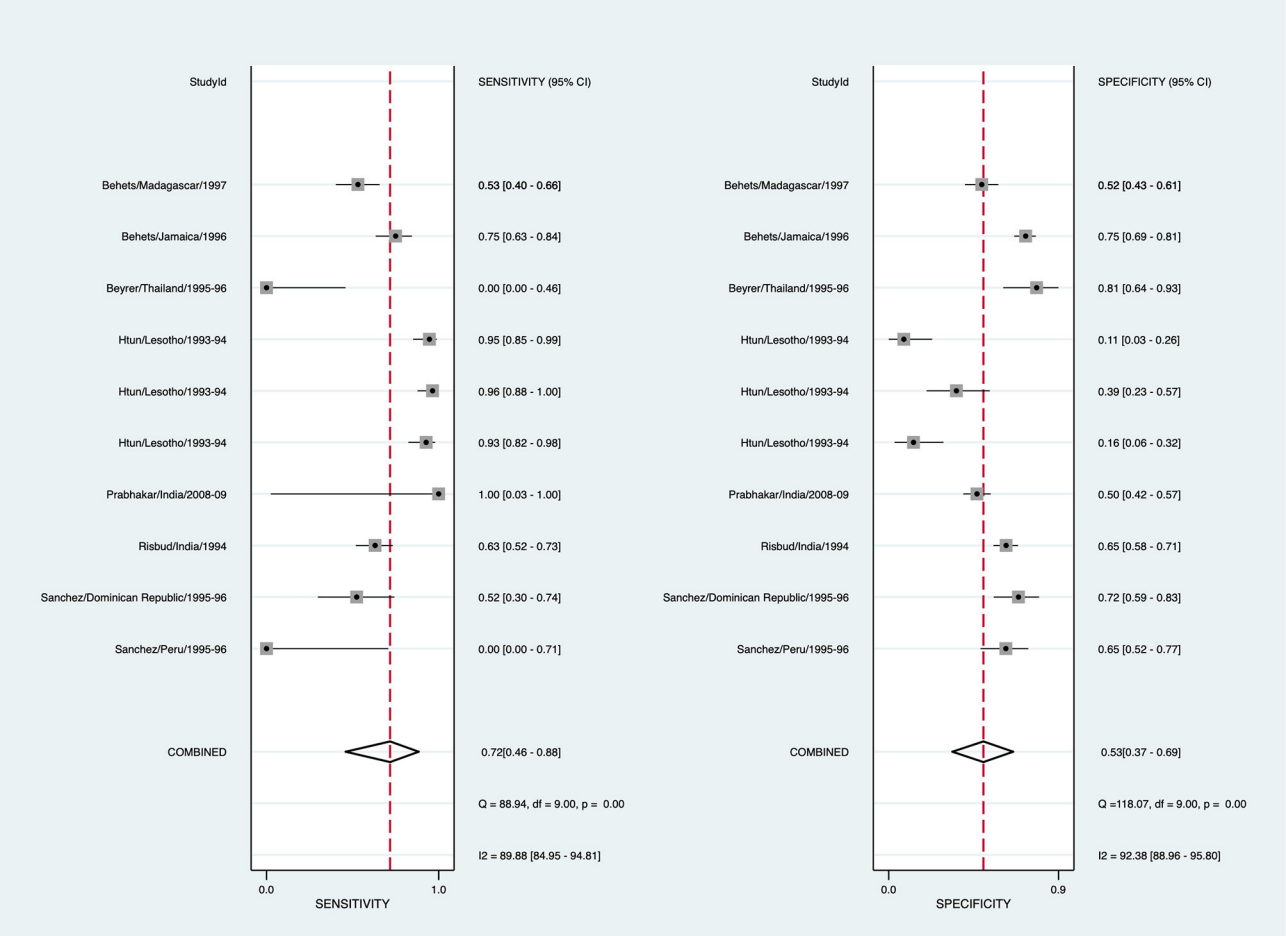
Forest plot of the sensitivity and specificity of clinical diagnosis to detect chancroid in genital ulcer disease.

To arrive at a presumptive diagnosis of *Haemophilus ducreyi* based on clinical evaluation, 13/29 studies provided 18 estimates, and 7 studies provided 10 estimates for pooling (Supplementary Table 6). The pooled sensitivity for detecting chancroid using clinical diagnosis was 71.9% (95% CI: 45.9–88.5, *I*^2^ = 89.9%), and pooled specificity was 53.1% (95% CI: 36.6–68.9, *I*^2^ = 92.4%) (Figure 4). The diagnostic odds ratio was 2.89 (95% CI: 1.34–6.23). The positive likelihood ratio was 1.53 (95% CI: 1.19–1.97), and the negative likelihood ratio was 0.53 (95% CI: 0.29–0.97). The inverse negative likelihood ratio was 1.89 (95% CI: 1.03–3.45). The number of missed and overtreated cases over different background prevalence is in Supplementary Table 7. The summary receiver operating curve is in Supplementary Figure 3.

The diagnostic accuracy of using clinical diagnosis to identify herpes translates to 17 missed cases, and eight treated unnecessarily in a population with a 30% prevalence of herpes (Supplementary Table 8). The certainty of the evidence is high because the studies were at low risk of bias (Supplementary Table 9), the statistical testing that was undertaken suggested no evidence of publication bias (Supplementary Figure 4), and although there was high heterogeneity, the confidence intervals around the estimates were not wide.

The test accuracy of using clinical diagnosis to identify syphilis translates to 3 missed cases, and 27 treated unnecessarily in a population with a 5% baseline prevalence of syphilis (Supplementary Table 10). The certainty of the evidence is moderate because there was high heterogeneity leading to wide confidence intervals around the estimates, but the risk of bias of the included studies was low (Supplementary Table 9), and the statistical testing that was undertaken suggested no evidence of publication bias (Supplementary Figure 5).

The test accuracy of using clinical diagnosis to identify chancroid translates to 4 missed cases, and 45 treated unnecessarily in a population with a 5% baseline prevalence of chancroid (Supplementary Table 11). The certainty of the evidence is moderate because there was high heterogeneity leading to wide confidence intervals around the estimates, but the risk of bias of the included studies was low (Supplementary Table 9), and the statistical testing that was undertaken suggested no evidence of publication bias (Supplementary Figure 6).

Meta-regression (Supplementary Tables 15–20) showed that country income level was significantly associated with the sensitivity and specificity of using clinical diagnosis to detect herpes and syphilis, and the publication year for the specificity of using clinical diagnosis to detect herpes and syphilis. The variables (country income level, year of publication, and recruitment site) included in the multivariable meta-regression models collectively explained up to 80% of the between-study variance for sensitivity and specificity across all aetiologies.

## Discussion

This systematic review and meta-analysis consolidate the published evidence of the effectiveness of syndromic management of GUD. The current WHO GUD algorithm contains decision points where clinical decisions must be made to offer HSV management or treat syphilis and chancroid (4). Our findings demonstrate that attempts to distinguish major pathogens clinically are unreliable—with low pooled sensitivity and relatively high pooled specificity. This could be attributed to the similarities between the clinical appearances of the various causes of GUD, the presence of mixed infections and atypical presentations (such as atypical clinical ulceration due to long-standing disease) (20–22). However, we also observed heterogeneity in the sensitivities and specificities, which might indicate that the accuracy of clinical diagnosis may depend on factors beyond those assessed in our meta-regression, such as clinician skill and experience, or other practise-specific factors. Similarly, the diagnostic accuracy of a pure syndromic approach to GUD, also found poor sensitivities in detecting the aetiological causes of GUD (Supplementary Tables 12–14).

Despite the poor diagnostic accuracy of clinical diagnosis methods and syndromic approaches to GUD management, these approaches still have a place in many settings. This is in light of various shortcomings associated with aetiological laboratory diagnosis methods. First, skilled personnel and quality assured laboratory equipment are needed. For example, multiplex PCR, one of the diagnostic tests for ulcers, is costly, technically sophisticated, time-consuming and rarely accessible in resource-limited settings. Second, as results for aetiological laboratory diagnosis methods are often unavailable the same day, additional patient follow up may be required to discuss results and subsequent treatment. However, this can be challenging when there is often high loss to follow up rates in the management of STIs due to poor awareness and fear of social stigma attached with STIs (23). Finally, there are limitations with currently available laboratory aetiological diagnostic tools. For example, syphilis serology tests may be falsely negative in early syphilitic ulcers (24). Similarly, darkfield microscopy may be relatively insensitive for the detection of syphilis (24), although it is still used in specialist sexual health services today. These missed cases may result in significant morbidity, mortality and onward transmission of the disease.

Therefore, in the absence of access to molecular-based testing and/or results on the same day, having a syndromic management algorithm is preferable to no management for people presenting with GUD (25). A syndromic management algorithm enables healthcare providers to come to a probable diagnosis and provide treatment on the same day without special skills or sophisticated and costly laboratory testing. In several countries, it was found that syndromic management offered adequate treatment for patients with GUD (25, 26). The syndromic management approach has been reported to be cost-saving or cost-effective and thus more easily implemented in settings where funding is an issue. However, most economic evaluations were performed more than 15 years ago in Peru (27), China (28), Taiwan (29) and sub-Saharan Africa (30). There is an urgent need for updated economic evaluations for the syndromic management of GUD.

When using syndromic management of GUD, one must be mindful of its limitations. In a case series of patients with GUD in India, a significant proportion of cases (34%, 24/71) classified and treated HSV using syndromic management were found to be VDRL positive (and would have been left untreated if they had not had lab testing) (31). Another example was from China, where a modified WHO GUD algorithm was used, i.e., patients with either recurrent, vesicular or painful ulcers were considered to have herpes (32). Overall, this led to an overdiagnosis of herpes and underdiagnosis of syphilis as not all painful or recurrent ulcers necessarily indicated an aetiology of herpes, and this excluded the presence of syphilis. This highlights the difficulties in algorithms requiring clinical judgment to distinguish the likely pathogen(s); syphilitic chancres and HSV ulcers can present atypically (33, 34). Overdiagnosis may be an issue in communities where STIs are still heavily associated with societal stigma (35). A wrong diagnosis may put the patient through unnecessary stress and can perpetuate STI-related stigma. There is also a potential for the loss of confidence in the healthcare system amongst patients inappropriately managed, which might discourage them from seeking medical help for future STI-related presentations and allowing undiagnosed STIs to spread throughout the community. Another critical point is that the syndromic approach may not significantly reduce the prevalence of STIs at a population level due to most STIs being asymptomatic. For example, in Kenya and Peru, implementation of the syndromic approach did not lead to significant reductions in the prevalence of STIs (36, 37).

The acceptability of syndromic management is mixed among clinicians [acceptable in Peru (36), Pakistan (38), but not in Namibia (39), and Karachi (40)] and patients [acceptable in Tanzania (41), but not in Rwanda (42)]. A study where standardised simulated patients visited pharmacies in Tanzania reported challenges for adequately managing GUD syndromes (43). Pharmacy staff in The Gambia were willing to offer syndromic management, but none of the simulated patients with GUD would be treated appropriately (44). There are also reports of inadequately following syndromic management guidelines. For example, interviews of healthcare workers from 240 healthcare facilities in six countries in West Africa found suboptimal STI management with effective treatment given to only 14% of patients (45). Although there was a high volume of potential STIs seen in community pharmacies, none of the 85 head pharmacists from South Africa correctly identified the treatment for genital ulcers (46).

Our findings have implications for future algorithms of syndromic management of GUD. As our meta-analysis noted that the clinical diagnosis to distinguish between herpes and syphilis is not sensitive enough, new GUD algorithms should consider offering management for syphilis and herpes for any patient with GUD, particularly in settings where syphilis prevalence is high. The trade-off between overtreatment and not missing cases may be appropriate in settings and populations where missing syphilis cases must be avoided (e.g., pregnant women). It can also be argued that although overtreatment can contribute to antimicrobial resistance development, there is less concern for syphilis or herpes; to date, there has been no documented syphilis resistance to penicillin. Second, strategic use of Point of Care Testing (POCT) within the algorithm can improve diagnostic accuracy. For example, when GUD diagnostic algorithms in Rwanda included an RPR test, overtreatment for syphilis reduced from 72.1 to 7.7% (25). There are also qualitative real-time PCR (qPCR) assays for detecting HSV-1, HSV-2, Varicella zoster virus (VZV), and *Treponema pallidum* with excellent sensitivities and specificities (47). However, the cost-effectiveness of incorporating PCR into GUD algorithms has not yet been evaluated for use in resource-poor countries. Third, as the aetiology of GUD can change over time [e.g., disappearance of chancroid as an important cause of GUD for many parts of the world (11) and rise of HSV as the dominant aetiology (12)], periodic monitoring of the underlying aetiology of GUD can help provide locally relevant guidance for syndromic management. In addition, the routine treatment for other less common causes of GUD (e.g., lymphogranuloma venereum) may be considered based on local STI prevalence rates.

Our findings should be read in light of several limitations. First, among the included studies in our meta-analysis, most participants were recruited from sexual health clinics, which means the results may not apply to other populations or settings where clinicians may be less skilled in managing GUD. Second, as studies were cross-sectional, we could not assess the temporal association between symptoms and STIs. Third, reference laboratory tests in studies included in the meta-analysis were not similar in their specificities and sensitivities, potentially contributing to the heterogeneity noted in our meta-analyses. For example, while the culture of chancroid is highly specific, its sensitivity is poor (48). HSV specific serologic assays can also have variable sensitivity and specificity, and may not be the temporal cause of GUD (49). Syphilis serology tests without concurrent molecular testing may miss early syphilis. Another factor to consider is how untested pathogens (e.g., lymphogranuloma venereum) could cause anogenital ulceration, even though they are less common. Last, most included studies were published before 2010. If there has been improvements in clinicians' ability to determine the aetiology of GUD through better training, our estimates may be an underestimate of the accuracy of this approach. However, there is evidence of the challenge to determine the aetiology of GUD using a clinical approach, even for highly trained sexual health specialist in a high-income country (50).

Algorithms requiring a clinical diagnosis to determine and treat the aetiology of GUD have poor sensitivities for syphilis and HSV, resulting in significant numbers of missed cases. A pure syndromic approach to GUD can also miss asymptomatic STIs or misdiagnose non-STI related GUD as STIs. These results can inform guideline recommendations by weighing the benefits, harms, costs, acceptability and feasibility of implementing algorithms for GUD. Moving forward, there is an urgent need to improve access to affordable and efficient diagnostics (e.g., POCTs) to be incorporated into GUD algorithms to better guide appropriate management.

## Data Availability Statement

The original contributions presented in the study are included in the article/Supplementary Material, further inquiries can be directed to the corresponding author/s.

## Author Contributions

JO, TW, PM, and NS designed the research study. JF conducted the search strategy. AL, ET, and JO conducted the screening, data extraction, and wrote the first draft of the paper. JO, AL, ET, RO-A, NS, and EC analysed the data. PM and RO-A interpreted the data. All authors have read and approved the final manuscript.

## Funding

The World Health Organization funded the study and helped with the study design, analysis, interpretation of data, writing of the report, and the decision to submit the paper for publication. JO and EC were supported by the Australian National Health and Medical Research Council (NHMRC) Emerging Leadership Investigator Grant (GNT1193955 and GNT1172873).

## Conflict of Interest

RO-A is a current employee of Roche Products Ltd., UK. The views expressed in this article are his own and do not represent that of his employers. The remaining authors declare that the research was conducted in the absence of any commercial or financial relationships that could be construed as a potential conflict of interest.

## Publisher's Note

All claims expressed in this article are solely those of the authors and do not necessarily represent those of their affiliated organizations, or those of the publisher, the editors and the reviewers. Any product that may be evaluated in this article, or claim that may be made by its manufacturer, is not guaranteed or endorsed by the publisher.
