# Supplementary material for: The Diagnostic Accuracy of Syndromic Management for Genital Ulcer Disease: A Systematic Review and Meta-Analysis
Source: Front Med (Lausanne). 2022 Jan 3;8:806605. doi: 10.3389/fmed.2021.806605 (PMC8767480; doi:10.3389/fmed.2021.806605)
Supplement: Supplementary file 1 [file Data_Sheet_1.pdf]

TABLE OF CONTENTS

Supplementary Table 1. Detection of any STIs for genital ulcer syndrome

Supplementary Table 2. Comparing the accuracy of clinical diagnosis of herpes with aetiological diagnosis of herpes

Supplementary Table 3. Number of missed and overtreated cases if using clinical diagnosis to detect herpes in a cohort of 1000 individuals, over different background prevalence.

Supplementary Table 4. Comparing the accuracy of clinical diagnosis of syphilis with aetiological diagnosis of syphilis

Supplementary Table 5. Number of missed and overtreated cases if using clinical diagnosis to detect syphilis in a cohort of 1000 individuals, over different background prevalence.

Supplementary Table 6. Comparing the accuracy of the clinical diagnosis of chancroid with aetiological diagnosis of chancroid

Supplementary Table 7. Number of missed and overtreated cases if using the clinical diagnosis to detect chancroid in a cohort of 1000 individuals, over different background prevalence.

Supplementary Table 8. Consequences of using clinical diagnosis for herpes

Supplementary Table 9. Quality assessment of included studies using QUADAS checklist.

Supplementary Table 10. Consequences of using clinical diagnosis for syphilis

Supplementary Table 11. Consequences of using clinical diagnosis for chancroid

Supplementary Table 12. Comparing the accuracy of the presence of GUD with aetiological diagnosis of herpes

Supplementary Table 13. Comparing the accuracy of the presence of GUD with aetiological diagnosis of syphilis

Supplementary Table 14. Comparing the accuracy of the presence of GUD with aetiological diagnosis of chancroid

Supplementary Table 15. Meta-regression results of using clinical diagnosis to detect herpes

Supplementary Table 16. Meta-regression results of using clinical diagnosis to detect syphilis

Supplementary Table 17. Meta-regression results of using clinical diagnosis to detect chancroid

Supplementary Table 18. Meta-regression results of using the presence of genital ulcer disease to detect herpes

Supplementary Table 19. Meta-regression results of using the presence of genital ulcer disease to detect syphilis

Supplementary Table 20. Meta-regression results of using the presence of genital ulcer disease to detect chancroid

Supplementary Figure 1. Summary receiver operating characteristic curve for clinical diagnosis to detect herpes

Supplementary Figure 2. Summary receiver operating characteristic curve for clinical diagnosis of GUD to detect syphilis

Supplementary Figure 3. Summary receiver operating characteristic curve for clinical diagnosis of GUD to detect chancroid

Supplementary Figure 4. Deek's funnel plot asymmetry test using clinical diagnosis to detect herpes

Supplementary Figure 5. Deek's funnel plot asymmetry test using clinical diagnosis to detect syphilis

Supplementary Figure 6. Deek's funnel plot asymmetry test using clinical diagnosis to detect chancroid

Search strategy September 2019

Updated search strategy January 2021

MONASH University

APPENDIX

For detection of any STIs, 1 study provided 2 estimates for evaluating the accuracy of clinical diagnosis of any STIs for a population with GUD. There were too few studies to conduct a meta-analysis.

Supplementary Table 1. Detection of any STIs for genital ulcer syndrome

Study	Year of study	Country	Country income level	Sample size	Where recruited	Subpopulation	How is positive case defined	Pathogens	Diagnostics	True positive	False negative	False positive	True negative
Sanchez[1]	1995-6	Dominican Republic	Upper middle	81	General practice	100% male<br>100% GUD	Symptoms + examination[1]	HSV, TP, HD	M-PCR	13	12	28	28
Sanchez[1]	1995-6	Peru	Upper middle	63	General practice	100% male<br>100% GUD	Symptoms + examination[1]	HSV, TP, HD	M-PCR	2	7	29	25

[1] Clinicians made their clinical diagnosis based on the presence of vesicles, tenderness, induration, inguinal adenopathy, tender adenopathy.

For detecting herpes from a clinical diagnosis of herpes, 15 studies provided 20 estimates (Supplementary Table 3). We only pooled studies if they used PCR to confirm a diagnosis of herpes: 9 studies provided 12 estimates. The pooled sensitivity for detecting herpes using a syndromic management approach is 43.5% (95% CI: 26.2-62.4), and pooled specificity is 88.0% (95% CI: 67.0-96.3). The diagnostic odds ratio is 5.63 (95% CI: 3.04-10.43). The positive likelihood ratio is 3.62 (95% CI: 1.74-7.54), and negative likelihood ratio is 0.64 (95% CI: 0.53-0.78). The inverse negative likelihood ratio is 1.56 (95% CI: 1.28-1.90).

Supplementary Table 2. Comparing the accuracy of clinical diagnosis of herpes with aetiological diagnosis of herpes

Study	Year of study	Country	Country income level	Sample size	Where recruited	Subpopulation	How is positive case defined	Diagnostics	True positive	False negative	False positive	True negative
Behets[2]	1997	Madagascar	Low	196	Sexual health clinic	71% male	History and Clinical Examination[1]	M-PCR	0	19	2	175
Behets[3]	1996	Jamaica	Upper middle	304	Sexual Health clinic	83% male	Local flowchart[2]	M-PCR	85	73	24	122
Beyrer[4]	1995-6	Thailand	Upper middle	38	Sexual health clinic	79% female sex workers	History and Clinical Examination[1]	M-PCR	21	11	3	3
Bhavsar[5]	2011-12	India	Low middle	96	Hospital	79% male	Local flowchart[3]	Tzanck smear<br>IgM for HSV-2	33	0	38	25
Bogaerts[6]	1990-92	Rwanda	Low	395	General practice	63% male	WHO flowchart[4]	Cytopathic effect on Vero cells	4	85	4	302
Bogaerts[6]	1990-92	Rwanda	Low	395	General practice	63% male	WHO flowchart[5]	Cytopathic effect on Vero cells	4	85	4	302
Bogaerts[6]	1990-92	Rwanda	Low	395	General practice	63% male	Clinical approach, no flowchart[6]	Cytopathic effect on Vero cells	43	46	87	219

DiCarlo[7]	1990-1992	USA	High	220	Sexual health clinic	100% men	History + physical exam [7]	Culture	20	37	10	153
Hina[8]	2015-16	India	Low middle	96	Sexual health clinic	75% males	Clinical diagnosis [8]	Tzanck smears, HSV2-IgM	33	2	36	25
Htun[9]	1993-94	Lesotho	Low middle	92	Sexual health clinic		Clinical diagnosis [9]	MPCR	7	10	1	74
Htun[9]	1993-94	Lesotho	Low middle	92	Sexual health clinic		Clinical diagnosis [9]	MPCR	5	19	3	65
Htun[9]	1993-94	Lesotho	Low middle	92	Sexual health clinic		Clinical diagnosis [9]	MPCR	0	24	0	68
Prabhakar[10]	2008-9	India	Low middle	181	Sexual health clinic	100% male	Indian national guidelines for GUD [10]	M-PCR	59	31	37	54

Risbud[11]	1994	India	Low middle	302	Sexual health clinic		Clinical criteria with minimal lab support [11]	M-PCR	48	47	32	175
Sanchez[1]	1995-6	Dominican Republic	Upper middle	81	General practice	100% male	History + Examination [12]	M-PCR	19	16	10	36
Sanchez[1]	1995-6	Peru	Upper middle	63	General practice	100% male	History + Examination [12]	M-PCR	15	12	17	19
Wang[12]	1998-99	China	Upper middle	96	Sexual health clinic	52% males	Modified WHO flowchart [13]	M-PCR	25	8	36	27
Wang[13]	2000-1	China	Upper middle	227	Sexual health clinic	90% male	Modified WHO flowchart[14]	M-PCR	49	22	78	78
Fast[14]	1980	Kenya	Low middle	70	"Special treatment clinic"	100% male	Examination [15]	Culture	3	3	1	63
Dangor[15]	Unclear	South Africa	Upper middle	210	Hospital	100% male	Clinical diagnosis[16]	Culture	5	2	21	182

[1] Clinical diagnosis based on history and physical examination.

[2] Guided by the Jamaican practical case management of common STD syndromes.

[3] Guided by the NACO guidelines of enhanced syndromic management. Herpes = painful vesicles/ulcers, single or multiple. Syphilis = painless ulcer with shotty lymph node. LGV = transient ulcer with inguinal lymph nodes. Chancroid = painful ulcer sometimes single giant ulcer associated with painful bubo. Suggests treatment for HSV only if vesicles or multiple painful ulcers. If not, treat for syphilis and chancroid.

[4] WHO flowchart 1. Herpes = Vesicular lesion present or history of vesicular lesions, often recurrent. Syphilis and chancroid = not diagnosed as herpes.

[5] WHO flowchart 2. Herpes = Vesicular lesion present or history of vesicular lesions, often recurrent. Syphilis = additional use of serological test for syphilis and/or darkfield microscopy. Chancroid = if not treated for herpes or syphilis.

[6] Chancroid = invasive ulcers. Primary syphilis = non-invasive ulcers. Herpes = Vesicles, history of recurrences, superficial ulcers (erosions).

[7] Clinical diagnosis based on history (using a standard interview form) and physical examination conducted by study nurses using objective criteria for the quantitative assessment of the ulcers.

[8] Method of clinical diagnosis was not stated.

[9] Clinical diagnoses were made by using ‘predefined criteria’ at the initial visit.

[10] Indian National Guidelines. Herpes = presence of vesicles or multiple painful ulcers with or without a history of recurrence. Non-herpetic = treated as per the national GUD algorithm.

[11] Primary syphilis = single painless ulcer with positive dark field examination of ulcer exudate. Secondary syphilis = multiple painless ulcers with positive RPR and positive darkfield microscopy. Chancroid = Single or multiple painful ulcers with a dirty base and darkfield examination was negative. Genital herpes = Multiple vesicles or small clusters of ulcers with negative darkfield examination. LGV = painful inguinal bubos, negative syphilis serology and with or without a history of a small shallow transient ulcer. Granuloma inguinale = painless deeply red, sharply defined ulcers which bled on touch and with negative syphilis serologies

[12] Standardised histories and examinations done. The characteristics of the lesions, presence of adenopathy and their clinical impressions of the probable cause of the lesions were noted. The results of clinical examinations for genital ulcers and vesicles and M-PCR tests for T pallidum, H ducreyi and HSV in genital lesions were used to retrospectively assess potential sensitivity, specificity and PPV of (in terms of treatment of chancroid and syphilis) of a published WHO flow chart algorithm for syndromic management of genital ulcers

[13] Method of clinical diagnosis not clear.

[14] Method of clinical diagnosis not clear.

[15] Chancroid = single or multiple genital ulcers with or without painful and/or suppurative inguinal buboes; ulcer is soft, nonvesicular, painful, invasive and purulent, with irregular and undermined edges. Primary syphilis = indurated, painless ulcer; bilateral, nontender, nonsuppurative inguinal adenopathy. Genital herpes = multiple vesicular lesions, often accompanied by tender inguinal adenopathy; vesicles may become secondarily infected and/or ulcerated. Lymphogranuloma venereum = transient genital lesion followed by bilateral, indolent, nontender adenopathy; multilocular suppuration and fistula formation may occur

[16] Chancroid = painful, irregular deep lesions with raised red margins and soft purulent bases. Primary syphilis = non-tender, relatively avascular, indurated, with smooth non-purulent base. Genital herpes = small, superficial, painful, with or without vesicles, history of recurrence. Donovanosis = exuberant, velvety red, non-painful. Lymphogranuloma venereum = significant, painful regional lymph node.

Supplementary Table 3. Number of missed and overtreated cases if using clinical diagnosis to detect herpes in a cohort of 1000 individuals, over different background prevalence.

Prevalence	Sensitivity	Specificity	PPV	NPV	Number of cases	Missed cases	False Positive (Overtreated)
0.05	0.435	0.88	0.160	0.967	50	28	114
0.1	0.435	0.88	0.287	0.933	100	57	108
0.15	0.435	0.88	0.390	0.898	150	85	102
0.2	0.435	0.88	0.475	0.862	200	113	96
0.25	0.435	0.88	0.547	0.824	250	141	90
0.3	0.435	0.88	0.608	0.784	300	170	84
0.35	0.435	0.88	0.661	0.743	350	198	78
0.4	0.435	0.88	0.707	0.700	400	226	72
0.45	0.435	0.88	0.748	0.656	450	254	66
0.5	0.435	0.88	0.784	0.609	500	283	60
0.55	0.435	0.88	0.816	0.560	550	311	54
0.6	0.435	0.88	0.845	0.509	600	339	48
0.65	0.435	0.88	0.871	0.456	650	367	42
0.7	0.435	0.88	0.894	0.400	700	396	36
0.75	0.435	0.88	0.916	0.342	750	424	30
0.8	0.435	0.88	0.935	0.280	800	452	24
0.85	0.435	0.88	0.954	0.216	850	480	18
0.9	0.435	0.88	0.970	0.148	900	509	12
0.95	0.435	0.88	0.986	0.076	950	537	6

1	0.435	0.88	1.000	0.000	1000	565	0

MONASH University

For detection of syphilis using clinical diagnosis of syphilis among individuals with GUD, 15 studies provided 22 estimates (Supplementary Table 4). We pooled studies which used PCR: 8 studies provided 11 estimates. The pooled sensitivity for detecting syphilis is 72.8% (95% CI: 51.4-87.1), and pooled specificity is 76.4% (95% CI: 45.4-92.6). The diagnostic odds ratio is 8.65 (95% CI: 3.20-23.38). The positive likelihood ratio is 3.08 (95% CI: 1.27-7.46), and negative likelihood ratio is 0.36 (95% CI: 0.21-0.60). The inverse of the negative likelihood ratio is 2.81 (95% CI: 1.68-4.69).

Supplementary Table 4. Comparing the accuracy of clinical diagnosis of syphilis with aetiological diagnosis of syphilis

Study	Year of study	Country	Country income level	Sample size	Where recruited	Subpopulation	How is positive case defined	Diagnostics	True positive	False negative	False positive	True negative
Behets[2]	1997	Madagascar	Low	196	Sexual health clinic	71% male	Clinical diagnosis[1]	M-PCR	52	4	112	28
Behets[3]	1996	Jamaica	Upper middle	304	Sexual Health clinic	83% male	Clinical diagnosis[2]	M-PCR	21	10	24	249
Beyrer[4]	1995-6	Thailand	Upper middle	38	Sexual health clinic	79% female sex workers	Clinical diagnosis[1]	M-PCR	0	1	1	36
Bhavsar[5]	2011-12	India	Low middle	96	Hospital	79% male	Clinical diagnosis[3]	VDRL, TPHA	19	24	1	52
Bogaerts[6]	1990-92	Rwanda	Low	395	General practice	63% male	WHO flowchart[4]	RPR, TPHA, Darkfield microscopy	108	2	279	6
Bogaerts[6]	1990-92	Rwanda	Low	395	General practice	63% male	WHO flowchart[5]	RPR, TPHA, Darkfield microscopy	107	3	9	276

Bogaerts[6]	1990-92	Rwanda	Low	395	General practice	63% male	Clinical diagnosis[6]	RPR, TPHA, Darkfield microscopy	20	90	31	254
DiCarlo[7]	1990-1992	USA	High	220	Sexual health clinic	100% men	Clinical diagnosis[7]	Darkfield microscopy	14	31	3	172
Hanson[16]	1996	Zambia	Low middle	95	Hospital	100% Male	Clinical diagnosis[8]	Darkfield microscopy, RPR, TPHA	24	17	14	40
Hanson[16]	1996	Zambia	Low middle	131	Hospital	100% Female	Clinical diagnosis[8]	Darkfield microscopy, RPR, TPHA	14	22	12	83
Htun[9]	1993-94	Lesotho	Low middle	92	Sexual health clinic		Clinical diagnosis[1]	MPCR, RPR, FTA-Abs	5	18	1	68
Htun[9]	1993-94	Lesotho	Low middle	92	Sexual health clinic		Clinical diagnosis[1]	MPCR, RPR, FTA-Abs	30	4	52	6

Htun[9]	1993-94	Lesotho	Low middle	92	Sexual health clinic		Clinical diagnosis[1]	MPCR, RPR, FTA-Abs	33	1	52	6
Ndinya-Achola[17]	1990-91	Kenya	Low middle	172	Primary care	47% males	History + Examination[9]	RPR	6	18	19	129
Prabhakar[10]	2008-9	India	Low middle	181	Sexual health clinic	100% male	Indian National Guidelines [10]	M-PCR	26	18	72	78
Sanchez[1]	1995-6	Dominican Republic	Upper middle	81	General practice	100% male	Clinical diagnosis[1]	M-PCR	2	2	11	66
Sanchez[1]	1995-6	Dominican Republic	Upper middle	63	General practice	100% male	History + Examination[11]	M-PCR	2	4	8	49
Wang[12]	1998-99	China	Upper middle	96	Sexual health clinic	100% had “STI symptoms”	Modified WHO Chart[1]	M-PCR, RPR, TPPA	18	5	12	61
Wang[13]	2000-1	China	Upper middle	227	Sexual health clinic	90% male	Modified WHO Chart[1]	M-PCR, Darkfield microscopy, RPR, TPPA	94	12	6	115
Fast[14]	1980	Kenya	Low middle	70	“Special treatment clinic”	100% male	Examination[12]	RPR, Darkfield microscopy	6	4	4	56

Dangor[15]	Unclear	South Africa	Upper middle	210	Hospital	100% male	Clinical diagnosis[13]	RPR, FTA-ABS, darkfield microscopy	22	3	25	160

[1] No clear description for clinical diagnosis.

[2] Guided by the Jamaican practical case management of common STD syndromes.

[3] Herpes = painful vesicles/ulcers, single or multiple. Syphilis = painless ulcer with shotty lymph node. LGV = transient ulcer with inguinal lymph nodes. Chancroid = painful ulcer sometimes single giant ulcer associated with painful bubo. Suggests treatment for HSV only if vesicles or multiple painful ulcers. If not, treat for syphilis and chancroid.

[4] WHO flowchart 1. Herpes = Vesicular lesion present or history of vesicular lesions, often recurrent. Syphilis and chancroid = not diagnosed as herpes.

[5] WHO flowchart 2. Herpes = Vesicular lesion present or history of vesicular lesions, often recurrent. Syphilis = additional use of serological test for syphilis and/or darkfield microscopy. Chancroid = if not treated for herpes or syphilis.

[6] Chancroid = invasive ulcers. Primary syphilis = non-invasive ulcers. Herpes = Vesicles, history of recurrences, superficial ulcers (erosions).

[7] Clinical diagnosis based on history (using a standard interview form) and physical examination conducted by study nurses using objective criteria for the quantitative assessment of the ulcers.

[8] Guidelines for treatment of STDs in primary care introduced by Zambian STD Control Programme in 1990. Standardised histories and examinations were done. Information collected about ulcers included their position, whether they were tender or not; superficial or deep; circumscribed or irregular; dirty or clean. The lymph nodes were characterized by size, confluence, consistency, tenderness and position as well as presence of Groove’s sign.

[9] History and physical examination conducted by clinical officer assigned to and trained for the project. Chancroid = painful, tender, soft ulcer with exudate, tender inguinal adenopathy. Syphilis = non-painful, non-tender, indurated ulcer without exudate, non- tender inguinal adenopathy. Herpes = painful, tender, soft ulcers with vesicles or pustules with or without tender inguinal adenopathy. LGV = painless transitory ulcer with prominent inguinal adenopathy

[10] Indian National Guidelines. Treatment of vesicles for HSV-2 and syphilis if rapid plasma reagin [RPR] positive or not received syphilis treatment recently; and for sores/ulcers without vesicles, treatment for syphilis, chancroid and HSV if HSV-2 seroprevalence is 93%. Herpes = presence of vesicles or multiple painful ulcers with or without a history of recurrence. Non-herpetic = treated as per the national GUD algorithm.

[11] Standardised histories and examinations done. The characteristics of the lesions, presence of adenopathy and their clinical impressions of the probable cause of the lesions were noted.

[12] Chancroid = single or multiple genital ulcers with or without painful and/or suppurative inguinal buboes; ulcer is soft, nonvesicular, painful, invasive and purulent, with irregular and undermined edges Primary syphilis = indurated, painless ulcer; bilateral, nontender, nonsuppurative inguinal adenopathy Genital herpes = multiple vesicular lesions, often accompanied by tender inguinal adenopathy; vesicles may become secondarily infected and/or ulcerated Lymphogranuloma venereum = transient genital lesion followed by bilateral, indolent, nontender adenopathy; multilocular suppuration and fistula formation may occur

[13] Chancroid = painful, irregular deep lesions with raised red margins and soft purulent bases Primary syphilis = non-tender, relatively avascular, indurated, with smooth non-purulent base Genital herpes = small, superficial, painful, with or without vesicles, history of recurrence Donovanosis = exuberant, velvety red, non-painful Lymphogranuloma venereum = significant, painful regional lymph nodes.

For detecting herpes from the presence of a genital ulcer, 7 studies provided 10 estimates. However, there were only two studies that used PCR, thus pooling was not appropriate.

Supplementary Table 5. Number of missed and overtreated cases if using clinical diagnosis to detect syphilis in a cohort of 1000 individuals, over different background prevalence.

Prevalence	Sensitivity	Specificity	PPV	NPV	Number of cases	Missed cases	False Positive (Overtreated)
0.05	0.728	0.764	0.140	0.982	50	14	224
0.1	0.728	0.764	0.255	0.962	100	27	212
0.15	0.728	0.764	0.352	0.941	150	41	201
0.2	0.728	0.764	0.435	0.918	200	54	189
0.25	0.728	0.764	0.507	0.894	250	68	177
0.3	0.728	0.764	0.569	0.868	300	82	165
0.35	0.728	0.764	0.624	0.839	350	95	153
0.4	0.728	0.764	0.673	0.808	400	109	142
0.45	0.728	0.764	0.716	0.774	450	122	130
0.5	0.728	0.764	0.755	0.737	500	136	118
0.55	0.728	0.764	0.790	0.697	550	150	106
0.6	0.728	0.764	0.822	0.652	600	163	94
0.65	0.728	0.764	0.851	0.602	650	177	83
0.7	0.728	0.764	0.878	0.546	700	190	71
0.75	0.728	0.764	0.902	0.484	750	204	59
0.8	0.728	0.764	0.925	0.413	800	218	47
0.85	0.728	0.764	0.946	0.331	850	231	35
0.9	0.728	0.764	0.965	0.238	900	245	24

0.95	0.728	0.764	0.983	0.129	950	258	12
1	0.728	0.764	1.000	0.000	1000	272	0

For detection of chancroid, 13 studies provided 18 estimates (Supplementary Table 6). We pooled studies which used PCR: 7 studies provided 10 estimates. The pooled sensitivity for detecting chancroid using clinical diagnosis was 71.9% (95% CI: 45.9-88.5), and pooled specificity was 53.1% (95% CI: 36.6-68.9). The diagnostic odds ratio was 2.89 (95% CI: 1.34-6.23). The positive likelihood ratio was 1.53 (95% CI: 1.19-1.97), and the negative likelihood ratio was 0.53 (95% CI: 0.29-0.97). The inverse negative likelihood ratio was 1.89 (95% CI: 1.03-3.45).

Supplementary Table 6. Comparing the accuracy of the clinical diagnosis of chancroid with aetiological diagnosis of chancroid

Study	Year of study	Country	Country income level	Sample size	Where recruited	Subpopulation	How is positive case defined	Diagnostics	True positive	False negative	False positive	True negative
Behets[2]	1997	Madagascar	Low	196	Sexual health clinic	71% male<br>100% GUD	History + Clinical Examination[1]	M-PCR	34	30	63	69
Behets[3]	1996	Jamaica	Upper middle	304	Sexual Health clinic	83% male<br>100% GUD	Local flowchart[2]	M-PCR	54	18	57	175
Beyrer[4]	1995-6	Thailand	Upper middle	38	Sexual health clinic	79% female sex workers<br>100% GUD	History + Clinical Examination[1]	M-PCR	0	6	6	26
Bhavsar[5]	2011-12	India	Low middle	96	Hospital	79% male<br>100% GUD	Local flowchart[3]	Gram stain	2	1	1	92
Bogaerts[6]	1990-92	Rwanda	Low	395	General practice	63% male<br>100% GUD	WHO flowchart[4]	Culture	115	0	272	8
Bogaerts[6]	1990-92	Rwanda	Low	395	General practice	63% male<br>100% GUD	WHO flowchart[5]	Culture	83	32	188	92
Bogaerts[6]	1990-92	Rwanda	Low	395	General practice	63% male<br>100% GUD	Clinical approach, no flowchart[6]	Culture	74	41	67	213
DiCarlo[7]	1990-1992	USA	High	220	Sexual health clinic	100% men<br>100% GUD	History + physical exam [7]	Culture	40	78	6	96

Htun[9]	1993-94	Lesotho	Low middle	92	Sexual health clinic	100% GUD	Clinical diagnosis [8]	MPCR	54	2	22	14
Htun[9]	1993-94	Lesotho	Low middle	92	Sexual health clinic	100% GUD	Clinical diagnosis [8]	MPCR	51	4	31	6
Htun[9]	1993-94	Lesotho	Low middle	92	Sexual health clinic	100% GUD	Clinical diagnosis [8]	MPCR	53	3	32	4
Ndinya-Achola[17]	1990-91	Kenya	Low middle	156	Primary care	47% males 100% GUD	History + Examination [9]	Culture	51	5	76	24
Prabhakar[10]	2008-9	India	Low middle	181	Sexual health clinic	100% male 100% GUD	Indian national guidelines for GUD [10]	M-PCR	59	31	37	54
Risbud[11]	1994	India	Low middle	302	Sexual health clinic	100% GUD	Clinical criteria with minimal lab support [11]	M-PCR	53	31	76	142
Sanchez[1]	1995-6	Dominican Republic	Upper middle	81	General practice	100% male 100% GUD	Symptoms + examination[12]	M-PCR	11	10	17	43

Sanchez[1]	1995-6	Peru	Upper middle	63	General practice	100% male<br>100% GUD	Symptoms + examination[12]	M-PCR	0	3	21	39
Fast[14]	1980	Kenya	Low middle	70	“Special treatment clinic”	100% male<br>100% GUD	Examination [13]	Culture	42	6	8	14
Dangor[15]	Unclear	South Africa	Upper middle	210	Hospital	100% GUD	Clinical diagnosis[14]		117	30	14	49

[1] Clinical diagnosis based on history and physical examination.

[2] Any genital epithelial disruption was considered to be a genital ulcer.

[3] Herpes = painful vesicles/ulcers, single or multiple. Syphilis = painless ulcer with shotty lymph node. LGV = transient ulcer with inguinal lymph nodes. Chancroid = painful ulcer sometimes single giant ulcer associated with painful bubo. Suggests treatment for HSV only if vesicles or multiple painful ulcers. If not, treat for syphilis and chancroid.

[4] WHO flowchart 1. Herpes = Vesicular lesion present or history of vesicular lesions, often recurrent. Syphilis and chancroid = not diagnosed as herpes.

[5] WHO flowchart 2. Herpes = Vesicular lesion present or history of vesicular lesions, often recurrent. Syphilis = additional use of serological test for syphilis and/or darkfield microscopy. Chancroid = if not treated for herpes or syphilis.

[6] Chancroid = invasive ulcers. Primary syphilis = non-invasive ulcers. Herpes = Vesicles, history of recurrences, superficial ulcers (erosions).

[7] Clinical diagnosis based on history (using a standard interview form) and physical examination conducted by study nurses using objective criteria for the quantitative assessment of the ulcers.

[8] Clinical diagnoses were made by using predefined criteria at the initial visit.

[9] History and physical examination conducted by clinical officer assigned to and trained for the project. Chancroid = painful, tender, soft ulcer with exudate, tender inguinal adenopathy. Syphilis = non-painful, non-tender, indurated ulcer without exudate, non- tender inguinal adenopathy. Herpes = painful, tender, soft ulcers with vesicles or pustules with or without tender inguinal adenopathy. LGV = painless transitory ulcer with prominent inguinal adenopathy

[10] Indian National Guidelines. Treatment of vesicles for HSV-2 and syphilis if rapid plasma reagin [RPR] positive or not received syphilis treatment recently; and for sores/ulcers without vesicles, treatment for syphilis, chancroid and HSV if HSV-2 seroprevalence is 93%. Herpes = presence of vesicles or multiple painful ulcers with or without a history of recurrence. Non-herpetic = treated as per the national GUD algorithm.

[11] Clinical criteria with minimal lab support. Primary syphilis = single painless ulcer with positive dark field examination of ulcer exudate. Secondary syphilis = multiple painless ulcers with positive RPR and positive darkfield microscopy. Chancroid = Single or multiple painful uclers with a dirty base and darkfield examination was negative. Genital herpes = Multiple vesicles or small clusters of ulcers with negative darkfield examination. LGV = painful inguinal bubos, negative syphilis serology and with or without a history of a small shallow transient ulcer. Granuloma inguinale = painless deeply red, sharply defined ulcers which bled on touch and with negative syphilis serologies

[12] Standardised histories and examinations done. The characteristics of the lesions, presence of adenopathy and their clinical impressions of the probable cause of the lesions were noted. The results of clinical examinations for genital ulcers and vesicles and M-PCR tests for T pallidum, H ducreyi and HSV in genital lesions were used to retrospectively assess potential sensitivity, specificity and PPV of (in terms of treatment of chancroid and syphilis) of a published WHO flow chart algorithm for syndromic management of genital ulcers

[13] Presumptive clinical diagnosis after initial examination. Chancroid = single or multiple genital ulcers with or without painful and/or suppurative inguinal buboes; ulcer is soft, nonvesicular, painful, invasive and purulent, with irregular and undermined edges Primary syphilis = indurated, painless ulcer; bilateral, nontender, nonsuppurative inguinal adenopathy Genital herpes = multiple vesicular lesions, often accompanied by tender inguinal adenopathy; vesicles may become secondarily infected and/or ulcerated Lymphogranuloma venereum = transient genital lesion followed by bilateral, indolent, nontender adenopathy; multilocular suppuration and fistula formation may occur

[14] Chancroid = painful, irregular deep lesions with raised red margins and soft purulent bases Primary syphilis = non-tender, relatively avascular, indurated, with smooth non-purulent base Genital herpes = small, superficial, painful, with or without vesicles, history of recurrence Donovanosis = exuberant, velvety red, non-painful Lymphogranuloma venereum = significant, painful regional lymph nodes.

Supplementary Table 7. Number of missed and overtreated cases if using the clinical diagnosis to detect chancroid in a cohort of 1000 individuals, over different background prevalence.

Prevalence	Sensitivity	Specificity	PPV	NPV	Number of cases	Missed cases	False Positive (Overtreated)
0.05	0.719	0.531	0.075	0.973	50	14	446
0.1	0.719	0.531	0.146	0.944	100	28	422
0.15	0.719	0.531	0.213	0.915	150	42	399
0.2	0.719	0.531	0.277	0.883	200	56	375
0.25	0.719	0.531	0.338	0.850	250	70	352
0.3	0.719	0.531	0.397	0.815	300	84	328
0.35	0.719	0.531	0.452	0.778	350	98	305
0.4	0.719	0.531	0.505	0.739	400	112	281
0.45	0.719	0.531	0.556	0.698	450	126	258
0.5	0.719	0.531	0.605	0.654	500	141	235
0.55	0.719	0.531	0.652	0.607	550	155	211
0.6	0.719	0.531	0.697	0.557	600	169	188
0.65	0.719	0.531	0.740	0.504	650	183	164
0.7	0.719	0.531	0.782	0.447	700	197	141
0.75	0.719	0.531	0.821	0.386	750	211	117
0.8	0.719	0.531	0.860	0.321	800	225	94
0.85	0.719	0.531	0.897	0.250	850	239	70
0.9	0.719	0.531	0.932	0.174	900	253	47
0.95	0.719	0.531	0.967	0.090	950	267	23
1	0.719	0.531	1.000	0.000	1000	281	0

MONASH University

Supplementary Table 8. Consequences of using clinical diagnosis for herpes

Pooled sensitivity : 0·44 (95% CI: 0·26 to 0·62) | Pooled specificity : 0·88 (95% CI: 0·67 to 0·96)

Test result	Number of results per 100 patients tested (95% CI)		Number of participants (studies)	Certainty of the Evidence (GRADE)
	Prevalence 30%	Prevalence 70%		
**Cases correctly identified with herpes and treated**	13 (8 to 19)	**31** (18 to 44)	1226 (9)	⊕⊕⊕⊕ HIGH [a]
**Cases of herpes missed**	17 (11 to 22)	40 (26 to 52)		
**Cases correctly identified without herpes and not treated**	62 (47 to 67)	26 (20 to 29)	1226 (9)	⊕⊕⊕⊕ HIGH [a]
**Cases unnecessarily treated for herpes**	8 (3 to 23)	4 (1 to 10)		

CI: Confidence interval

Explanations

a. Some heterogeneity but confidence intervals not wide.

Supplementary Table 9. Quality assessment of included studies using QUADAS checklist.

Study	Patient selection	Index Test	Reference standard		Flow and Timing
Behets[2]*	Low	Low	Low		Low
Behets[3]*	Low	Low	Low		Low
Beyrer[4]*	Low	Low	Low		Low
Bhavsar[5]*	Low	Low	Low	High[1]	Low
Bogaerts[6]*	Low	Low	Low	High[2]	Low
Choudhry[18]	Low	Low	Low	High[3]	Low
Clark[19]	Low	Low	Low		
Daly[20]	Low	Low	Low	High[4]	Low
Dangor[15]*	Low	Low	Low	High[5]	Low
Das[21]	Low	Low	Unclear		Low
Desai[22]	Low	Low	Low		Low
DiCarlo[7]*	Low	Low	Low	High[6]	Low
Fast[14]*	Low	Low	Low	High[5]	Low
Hanson[16]*	Low	Low	Low	High[3]	Low
Hina[8]*	Low	Low	High		Low

Htun[9]*	Low	Low	Low		Low
Liu[23]	Low	Low	Unclear		Low
Muralidhar[24]	Low	Low	Low		Low
Ndinya-Achola[17]*	Low	Low	High		Low
O'Farrell[25]	Low	Low	Low		Low
Otieno[26]	Low	Low	Low		High[6]
Prabhakar[10]*	Low	Low	Low		Low
Risbud[11]*	Low	Low	Low		Low
Sanchez[1]*	Low	Low	Low		Low
Shah[27]	Low	Low	Low	High[7]	Low
Shahesmaeili[28]	Low	Low	Low		Low
Tsai[29]	Low	Low	Low		Low
Wang[12]*	Low	Low	Unclear		Low
Wang[13]*	Low	Low	Low		Low
Yu[30]	Low	Low	Low		Low

[1] High risk for NG, HD, CG, HSV, Low risk for TP

[2] High risk for NG, HD, HSV, Low risk for TP

[3] High risk for CT, NG, HD, CG, HSV, Low risk for TP

[4] High risk for NG, Low risk for TP

[5] High risk for CT, HD, HSV, Low risk for TP

[6] High risk for HD, HSV, Low risk for TP

[7] High risk for HSV, Low risk for CT, NG, TP

* Studies that compare clinical diagnosis of GUD with laboratory confirmed STI diagnosis

Supplementary Table 10: Consequences of using clinical diagnosis for syphilis

Pooled sensitivity : 0·53 (95% CI: 0·23 to 0·81) | Pooled specificity : 0·72 (95% CI: 0·28 to 0·95)

**Test result**	**Number of results per 100 patients tested (95% CI)**		**Number of participants (studies)**	**Certainty of the Evidence (GRADE)**
	Prevalence 5%	Prevalence 10%		
**Cases correctly identified with syphilis and treated**	3 (1 to 4)	5 (2 to 8)	1462 (8)	⊕⊕⊕◯ MODERATE [a]
**Cases of syphilis missed**	2 (1 to 4)	5 (2 to 8)		
**Cases correctly identified without syphilis and not treated**	**69** (27 to 90)	65 (25 to 85)	1462 (8)	⊕⊕⊕◯ MODERATE [a]
**Cases unnecessarily treated for syphilis**	**27** (5 to 68)	25 (5 to 65)		

CI: Confidence interval

Explanations

a. Estimates from studies varied widely, meaning wide confidence intervals and then absolute effects.

MONASH University

Supplementary Table 11: Consequences of using clinical diagnosis for chancroid

Pooled sensitivity : 0·72 (95% CI: 0·46 to 0·89) | Pooled specificity : 0·53 (95% CI: 0·37 to 0·69)

Test result	Number of results per 100 patients tested (95% CI)		Number of participants (studies)	Certainty of the Evidence (GRADE)
	Prevalence 5%	Prevalence 10%		
**Cases correctly identified with chancroid and treated**	**4** (2 to 4)	7 (5 to 9)	1441 (7)	⊕⊕⊕◯ MODERATE [a]
**Cases of chancroid missed**	**1** (1 to 3)	**3** (1 to 5)		
**Cases correctly identified without chancroid and not treated**	**50** (35 to 66)	**48** (33 to 62)	1441 (7)	⊕⊕⊕◯ MODERATE [a]
**Cases unnecessarily treated for chancroid**	**45** (30 to 60)	42 (28 to 57)		

CI: Confidence interval

Explanations

a. Estimates from studies varied widely, meaning wide confidence intervals and then absolute effects.

For detection of herpes from presence of GUD, 7 studies provided 10 estimates (Supplementary Table 12). However, there were only two studies that used PCR, thus pooling was not appropriate.

Supplementary Table 12. Comparing the accuracy of the presence of GUD with aetiological diagnosis of herpes

Study	Year of study	Country	Country income level	Sample size	Where recruited	Subpopulation	How is positive case defined	Diagnostics	True positive	False negative	False positive	True negative
Choudhry[18]	2007-8	India	Low middle	300	Sexual health clinic	64% male<br><br>16% MSM	Syndromic approach algorithms recommended by National AIDS control organisation (NACO), India	Gram stain, HSV-IgM	57	12	3	228
Clark[19]	2003-5	Peru	Upper middle	3285	Community setting	73% heterosexual men<br><br>16% MSM	Interview	HSV2-Ab	78	770	162	2275
Liu[23]	2003	China	Upper middle	55	Sexual health clinic	100% male<br><br>14% GUD	WHO syndromic algorithms<br><br>Presence of ulcer	PCR	15	0	38	2

Muralidhar[24]	2013	India	Low middle	1208	Sexual health clinic	67% male<br><br>7.5% GUD	History + Examination	Giemsa stain, PCR, HSV2-IgM	76	6	5	1121
O’Farrell[25]	2007	South Africa	Upper middle	642	Sexual health clinic	100% heterosexuals<br><br>25.4% GUD	Symptoms + risk factors	HSV2-Ab	140	347	22	133
							Questionnaire + clinical examination					
Otieno[26]	2007-9	Kenya	Low middle	786	Enrolled in general population study	50% males<br><br>7% GUD	Interview + clinical examination	HSV2-IgG	0	14	14	796
Shah[27]	2008	El Salvador	Low middle	366	Hospital	100% females living with HIV	Self-reported symptoms	HSV-2 serology	61	262	5	38
Shah[27]	2008	El Salvador	Low middle	365	Hospital	10% males living with HIV	Self-reported symptoms	HSV-2 serology	55	7	234	69

Shah[27]	2008	El Salvador	Low middle	768	Hospital	FSW	Self-reported symptoms	HSV-2 serology	20	647	5	96
Shah[27]	2008	El Salvadro	Low middle	703	Hospital	MSM	Self-reported symptoms	HSV-2 serology	37	299	22	345

For detection of syphilis from presence of GUD, 12 studies provided 15 estimates (Supplementary Table 13). However, there were only three studies that used PCR, thus pooling was not appropriate.

Supplementary Table 13. Comparing the accuracy of the presence of GUD with aetiological diagnosis of syphilis

Study	Year of study	Country	Country income level	Sample size	Where recruited	Subpopulation	How is positive case defined	Diagnostics	True positive	False negative	False positive	True negative
Choudhry[18]	2007-8	India	Low middle	300	Sexual health clinic	64% male<br>16% MSM	Syndromic approach algorithms recommended by National AIDS control organisation (NACO), India	VDRL, TPHA	11	3	4	282
Clark[19]	2003-5	Peru	Upper middle	3285	Community setting	73% heterosexual men<br>16% MSM	History	RPR, TPPA	6	91	234	2954
Daly[20]	1989-91	Kenya	Low middle	4367	Family planning clinic	100% females	Clinical diagnosis	RPR	4	79	76	4208
Desai[22]	2000	India	Low middle	118	Sexual health clinic	100% FSW	Symptoms + Examination	RPR, TPHA	4	23	3	88

Liu[23]	2003	China	Upper middle	55	Sexual health clinic	100% male<br><br>14% GUD	WHO syndromic algorithms<br><br>Presence of ulcer	PCR, RPR, TPPA	13	0	40	2
Muralidhar[24]	2013	India	Low middle	90	Sexual health clinic	67% male<br><br>7.5% GUD	History + Clinical Examination	Darkfield microscopy, PCR, VDRL, TPHA, FTA-Abs	4	3	2	81
Shah[27]	2008	El Salvador	Low middle	366	Hospital	100% females living with HIV	Self-reported symptoms	RPR, TPPA	0	2	4	360
Shah[27]	2008	El Salvador	Low middle	365	Hospital	100% males living with HIV	Self-reported symptoms	RPR, TPPA	5	58	15	287
Shah[27]	2008	El Salvador	Low middle	768	Hospital	FSW	Self-reported symptoms	RPR, TPPA	1	16	24	727
Shah[27]	2008	El Salvador	Low middle	703	Hospital	MSM	Self-reported symptoms	RPR, TPPA	2	31	65	605
Shahesmaeili[28]	2015	Iran	Upper middle	1337	Community	100% FSW<br><br>3% GUD	Self-reported symptoms	Rapid tests – SD Bioline HIV/Syphilis Duo + Alere Syphilis RPR, EIA	0	5	40	1292
Tsai[29]	2008	Taiwan	High	138	Sexual health clinic	100% males<br><br>29% GUD	Clinical diagnosis	RPR, TPHA	26	7	86	19
O'Farrell[25]	2007	South Africa	Upper middle	645	Sexual health clinic	100% heterosexuals	History + clinical examination	RPR, TPPA	17	29	147	452

						25.4% GUD					
Otieno[26]	2007-9	Kenya	Low middle	824	Enrolled in general population study	50% males<br>7% GUD	History + clinical examination	RPR, TPPA	0	14	14	796
Yu[30]	2002-4	Taiwan	High	307	Sexual health clinic	100% male<br>11% GUD	Clinical diagnosis	M-PCR, RPR, TPHA	8	13	17	269

For detection of chancroid using GUD, 2 studies provided 2 estimates for pooling. We were unable to conduct a meta-analysis due to too few studies.

Supplementary Table 14. Comparing the accuracy of the presence of GUD with aetiological diagnosis of chancroid

Study	Year of study	Country	Country income level	Sample size	Where recruited	Subpopulation	How is positive case defined	Diagnostics	True positive	False negative	False positive	True negative
Liu[23]	2003	China	Upper middle	55	Sexual health clinic	100% male<br>14% GUD	WHO syndromic algorithms,<br>Presence of ulcer	PCR	0	0	53	2
Muralidhar[24]	2013	India	Low middle	90	Sexual health clinic	67% male<br>7.5% GUD	History + Examination.	Gram stain, culture, PCR	0	1	10	79

Supplementary Table 15. Meta-regression results of using clinical diagnosis to detect herpes

Variable	Univariable			Multivariable		
	β (95% CI)	P-value	Adjusted R^2	β (95% CI)	P-value	Adjusted R^2
Sensitivity						
**Country income level**			85.9%			80.1%
High (reference)						
Lower-Middle	1.5 (-0.4 to 3.3)	0.104		0.8 (-1.5 to 3.1)	0.435	
Upper-Middle	1.1 (-0.5 to 2.8)	0.162		0.9 (-1.1 to 2.8)	0.348	
Low	-2.6 (-4.9 to -0.2)	0.034		-2.7 (-5.5 to <0.01)	0.052	
**Publication**			30.9%			
Before 2000 (ref)						
2000 and after	-2.4 (-4.3 to -0.6)	0.013		-1.1 (-2.6 to 0.4)	0.117	
**Sexual health clinic**			-5.4%			
No (ref)						
Yes	0.26 (-1.5 to 2.0)	0.766		-0.2 (-1.5 to 1.2)	0.752	
Specificity						
**Country income level**			39.3%			68.2%
High (reference)						

Lower-Middle	-1.5 (-5.1 to 2.1)	0.373		0.2 (-3.0 to 3.4)	0.900
Upper-Middle	-2.0 (-5.5 to 1.5)	0.231		-1.7 (-4.5 to 1.0)	0.189
Low	1.6 (-2.5 to 5.6)	0.415		1.7 (-1.6 to 4.9)	0.269
**Publication**			22.9%		
Before 2000 (ref)					
2000 and after	2.4 (0.4 to 4.3)	0.023		2.5 (0.5 to 4.4)	0.019
**Sexual health clinic**			-2.9%		
No (ref)					
Yes	-0.5 (-2.3 to 1.3)	0.576		0.2 (-1.4 to 1.7)	0.780

Supplementary Table 16. Meta-regression results of using clinical diagnosis to detect syphilis

**Variable**	**Univariable**			**Multivariable**		
	β (95% CI)	P-value	Adjusted R^2	β (95% CI)	P-value	Adjusted
Sensitivity						
**Country income level**			73.0%			74.1%
High (reference)						
Lower-Middle	0.6 (-1.3 to 2.5)	0.524		0.2 (-2.0 to 2.3)	0.859	
Upper-Middle	1.8 (-0.1 to 3.8)	0.061		1.4 (-0.6 to 3.4)	0.149	
Low	4.0 (1.7 to 6.3)	0.003		3.9 (1.6 to 6.3)	0.004	
**Publication**			-7.0%			
Before 2000 (ref)						
2000 and after	-0.1 (-2.3 to 2.1)	0.952		-0.9 (-2.2 to 0.3)	0.134	
**Sexual health clinic**			-3.7%			
No (ref)						
Yes	0.4 (-1.1 to 2.0)	0.572		-0.1 (-1.3 to 1.1)	0.897	
Specificity						
**Country income level**			73.4%			66.6%
High (reference)						
Lower-Middle	-2.1 (-5.0 to 0.8)	0.141		-1.5 (-5.2 to 2.2)	0.379	
Upper-Middle	-1.9 (-4.7 to 1.0)	0.175		-1.6 (-5.0 to 1.9)	0.333	
Low	-6.6 (-9.8 to -3.4)	0.001		-6.3 (-10.1 to -2.5)	0.005	

**Publication**			-3.1%		
Before 2000 (ref)					
2000 and after	-1.0 (-3.9 to 1.9)	0.484		0.3 (-1.9 to 2.6)	0.742
**Sexual health clinic**			-4.5%		
No (ref)					
Yes	-0.4 (-2.5 to 1.6)	0.655		0.6 (-1.2 to 2.4)	0.486

Supplementary Table 17. Meta-regression results of using clinical diagnosis to detect chancroid

**Variable**	**Univariable**			**Multivariable**		
	β (95% CI)	P-value	Adjusted R^2	β (95% CI)	P-value	Adjusted
Sensitivity						
**Country income level**			21.0%			-5.4%
High (reference)						
Lower-Middle	2.7 (-0.5 to 6.0)	0.091		3.1 (-1.6 to 7.9)	0.157	
Upper-Middle	1.2 (-2.1 to 4.4)	0.440		0.7 (-3.8 to 5.3)	0.714	
Low	2.4 (-1.4 to 6.2)	0.183		2.6 (-2.2 to 7.5)	0.232	
**Publication**						
Before 2000 (ref)						
2000 and after	0.4 (-2.8 to 3.6)	0.800	-6.0%	1.6 (-2.6 to 5.9)	0.388	
**Sexual health clinic**						
No (ref)						
Yes	0.0 (-1.5 to 1.5)	0.968	-10.6%	-0.1 (-2.8 to 2.7)	0.968	
Specificity						
**Country income level**			31.8%			20.0%
High (reference)						
Lower-Middle	-2.3 (-6.3 to 1.6)	0.217		-3.1 (-8.2 to 2.1)	0.197	
Upper-Middle	-1.7 (-5.6 to 2.3)	0.363		-1.6 (-6.5 to 3.2)	0.439	
Low	-4.4 (-8.8 to 0.0)	0.049		-4.4 (-9.6 to 0.9)	0.089	

**Publication**			12.2%		
Before 2000 (ref)					
2000 and after	-2.5 (-5.7 to 0.7)	0.116		-2.2 (-6.2 to 1.8)	0.220
**Sexual health clinic**			-8.5%		
No (ref)					
Yes	-0.2 (-2.0 to 1.5)	0.779		0.1 (-1.7 to 11.5)	0.907

Supplementary Table 18. Meta-regression results of using the presence of genital ulcer disease to detect herpes`

Variable	Univariable			Multivariable		
	β (95% CI)	P-value	Adjusted R^2	β (95% CI)	P-value	Adjuste[illegible]
Sensitivity						
**Country income level**			-22.5%			60.6%
High (reference)						
Lower-Middle	0.0 (-5.6 to 5.5)	0.984		0.7 (-2.9 to 4.3)	0.604	
Upper-Middle	*			*		
Low	*			*		
**Publication**						
Before 2000 (ref)						
2000 and after	*			*		
**Sexual health clinic**						
No (ref)						
Yes	3.3 (0.3 to 6.3)	0.035	38.3%	3.9 (0.4 to 7.4)	0.038	
Specificity						
**Country income level**			38.6%			22.9%
High (reference)						
Lower-Middle	3.5 (-0.7 to 7.7)	0.086		3.5 (-1.7 to 8.6)	0.135	
Upper-Middle	*			*		

Low	*			*	
**Publication**					
Before 2000 (ref)					
2000 and after	*			*	
**Sexual health clinic**					
No (ref)					
Yes	0.2 (-3.7 to 4.1)	0.924	-14.5%	-0.4 (-5.5 to 4.7)	0.841

***** omitted because of collinearity

Supplementary Table 19. Meta-regression results of using the presence of genital ulcer disease to detect syphilis

Variable	Univariable			Multivariable		
	β (95% CI)	P-value	Adjusted R^2	β (95% CI)	P-value	Adjusted
Sensitivity						
**Country income level**			-12.3%			69.7%
High (reference)						
Lower-Middle	-1.7 (-5.2 to 1.9)	0.319		0.3 (-2.1 to 2.8)	0.752	
Upper-Middle	-1.2 (-5.1 to 2.6)	0.489		0.0 (-2.5 to 2.6)	0.964	
Low	*			*		
**Publication**			2.1%			
Before 2000 (ref)						
2000 and after	-1.4 (-4.3 to 1.5)	0.313		-3.1 (-5.7 to -0.6)	0.022	
**Sexual health clinic**			47.1%			
No (ref)						
Yes	2.4 (0.8 to 4.0)	0.007		3.2 (0.9 to 5.4)	0.012	
Specificity						
**Country income level**			34.8%			37.3%
High (reference)						
Lower-Middle	3.3 (-0.1 to 6.8)	0.057		2.4 (-1.7 to 6.5)	0.202	
Upper-Middle	0.6 (-3.1 to 4.2)	0.734		-0.5 (-4.5 to 3.6)	0.798	

Low	*			*	
**Publication**			0.6%		
Before 2000 (ref)					
2000 and after	1.4 (-1.7 to 4.5)	0.351		0.7 (-3.5 to 4.8)	0.721
**Sexual health clinic**			-2.3%		
No (ref)					
Yes	-1.1 (-3.4 to 1.1)	0.294		-2.0 (-5.1 to 1.1)	0.172

***** Omitted because of collinearity

Supplementary Figure 1. Summary receiver operating characteristic curve for clinical diagnosis to detect herpes

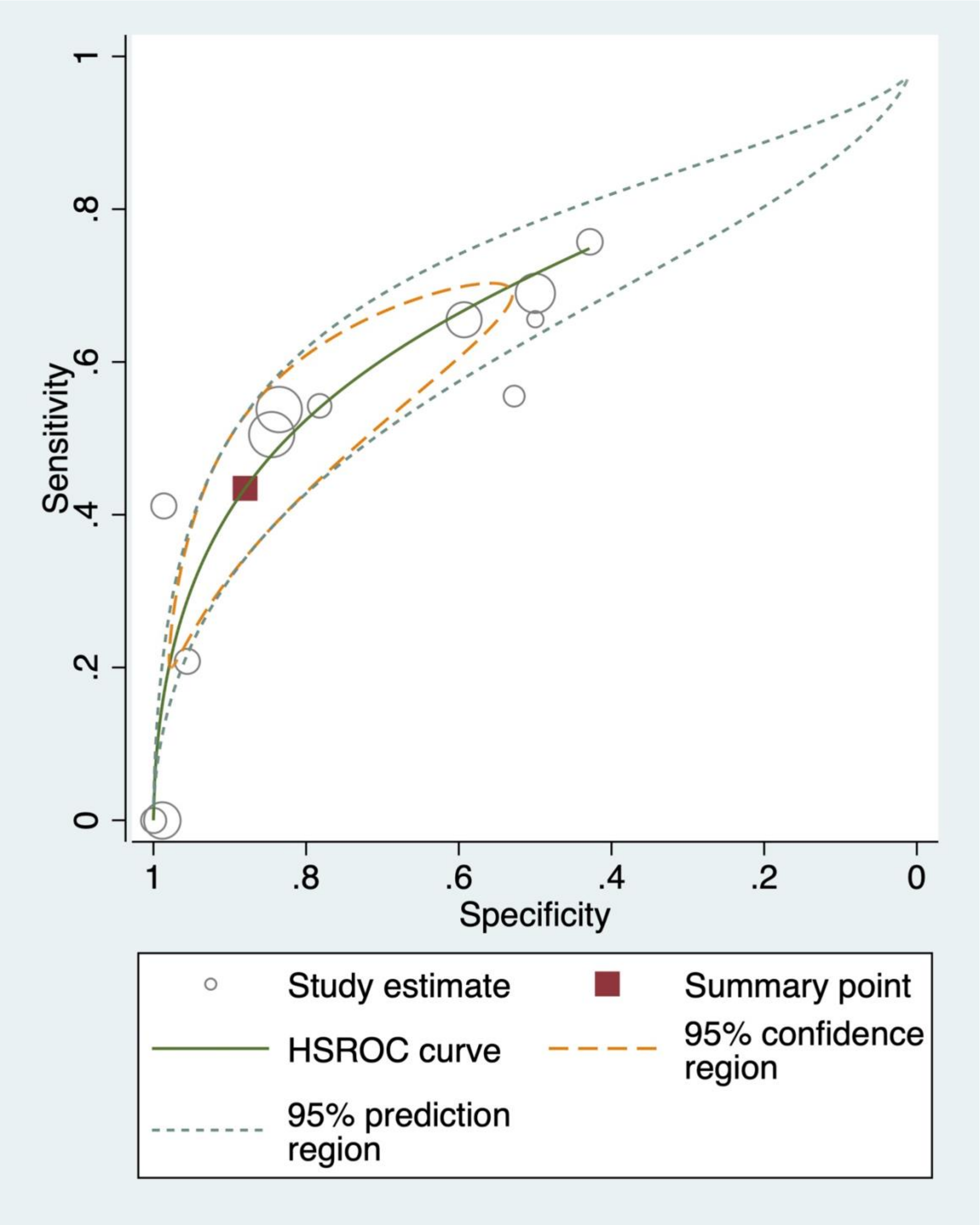

Supplementary Figure 2. Summary receiver operating characteristic curve for clinical diagnosis of GUD to detect syphilis

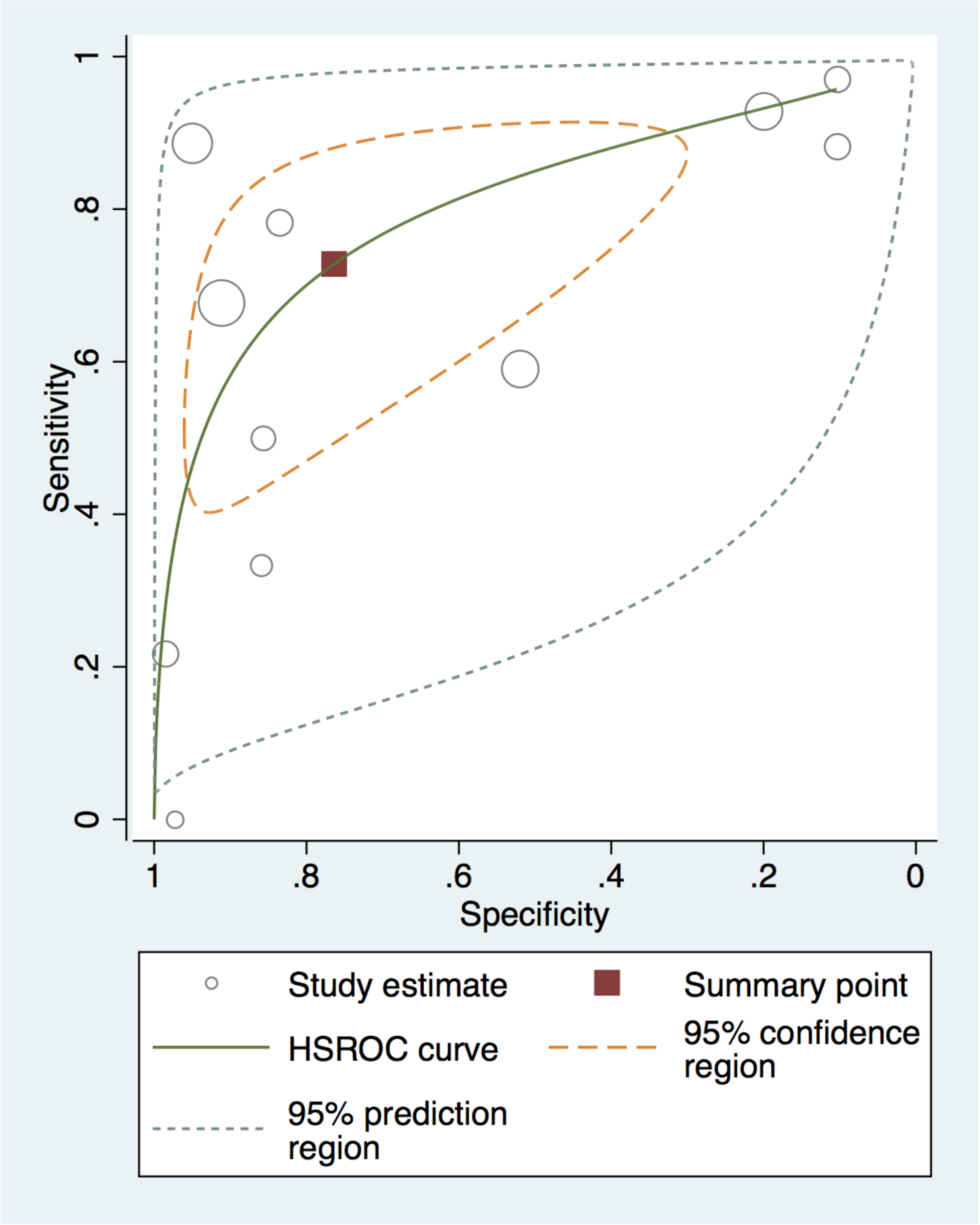

Supplementary Figure 3. Summary receiver operating characteristic curve for clinical diagnosis of GUD to detect chancroid

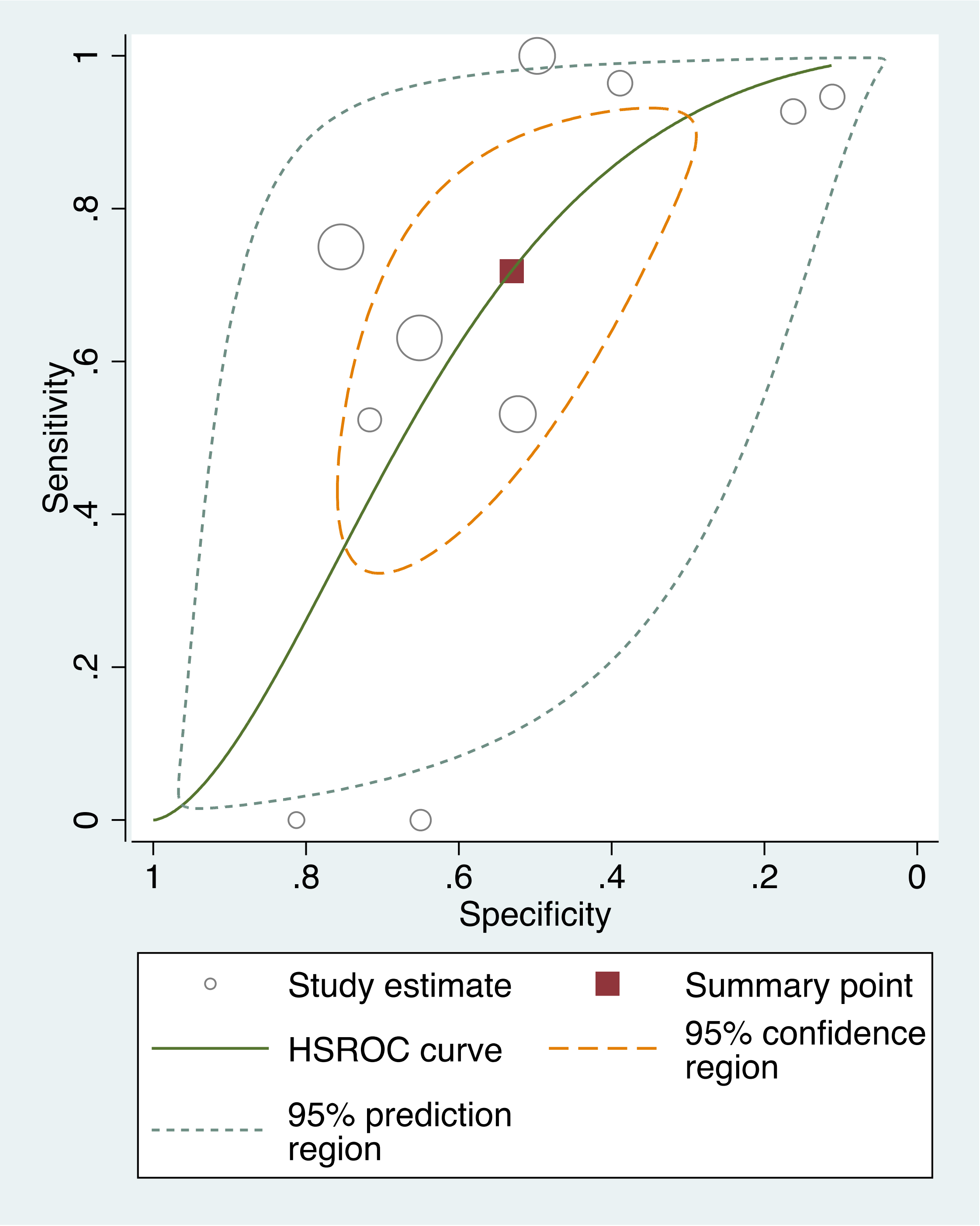

Supplementary Figure 4. Deek's funnel plot asymmetry test using clinical diagnosis to detect herpes

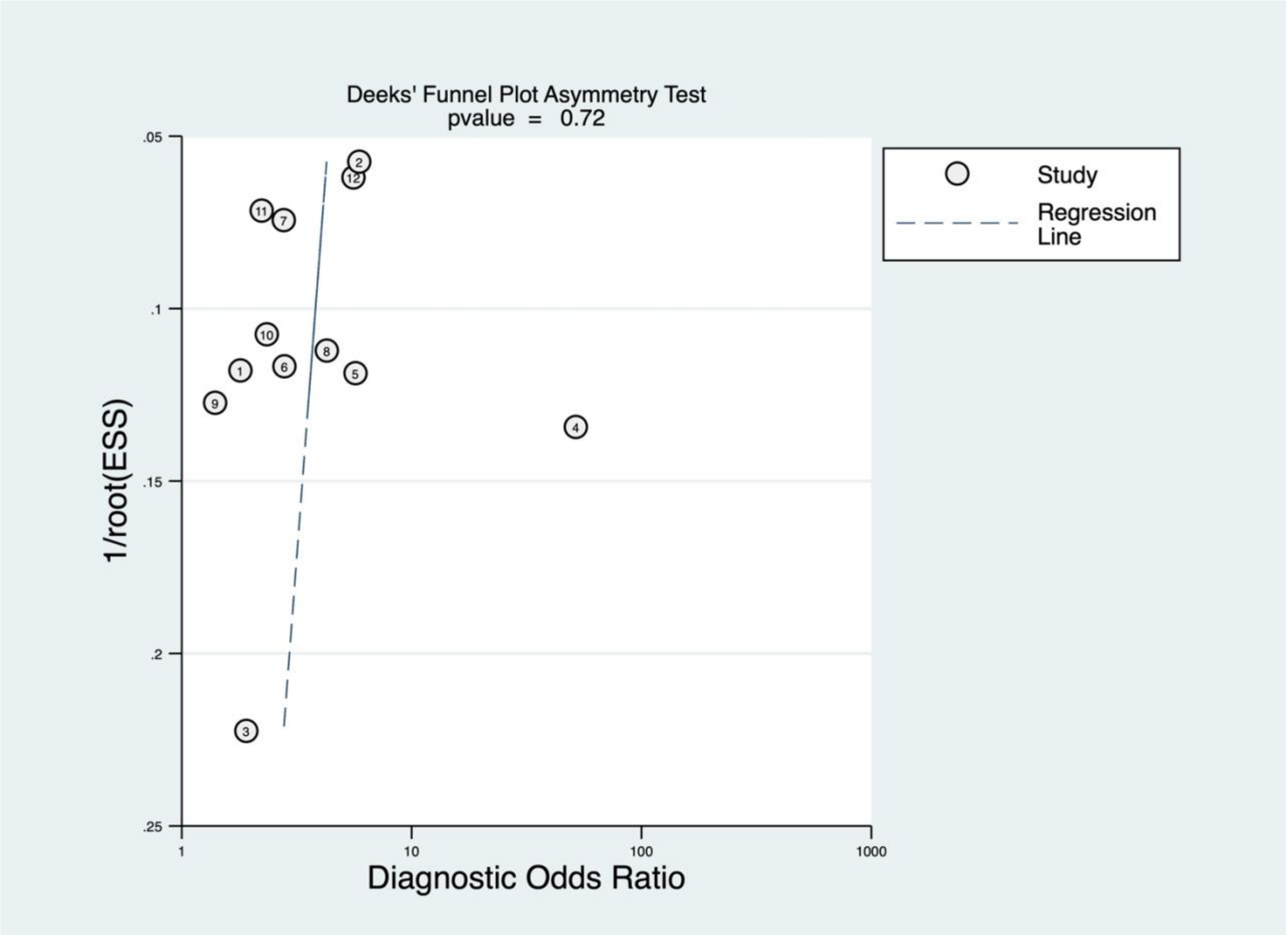

Supplementary Figure 5. Deek's funnel plot asymmetry test using clinical diagnosis to detect syphilis

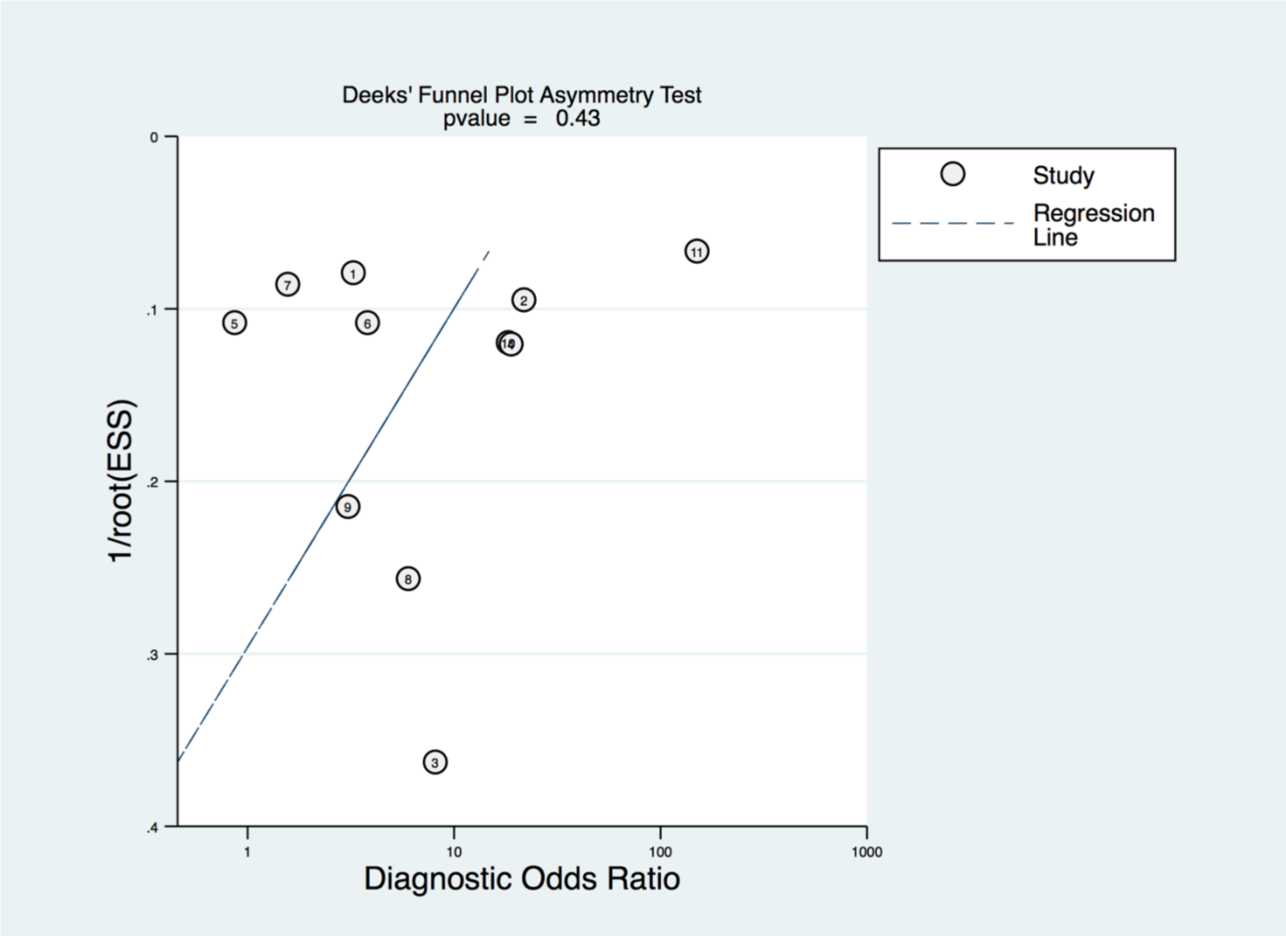

Supplementary Figure 6. Deek's funnel plot asymmetry test using clinical diagnosis to detect chancroid

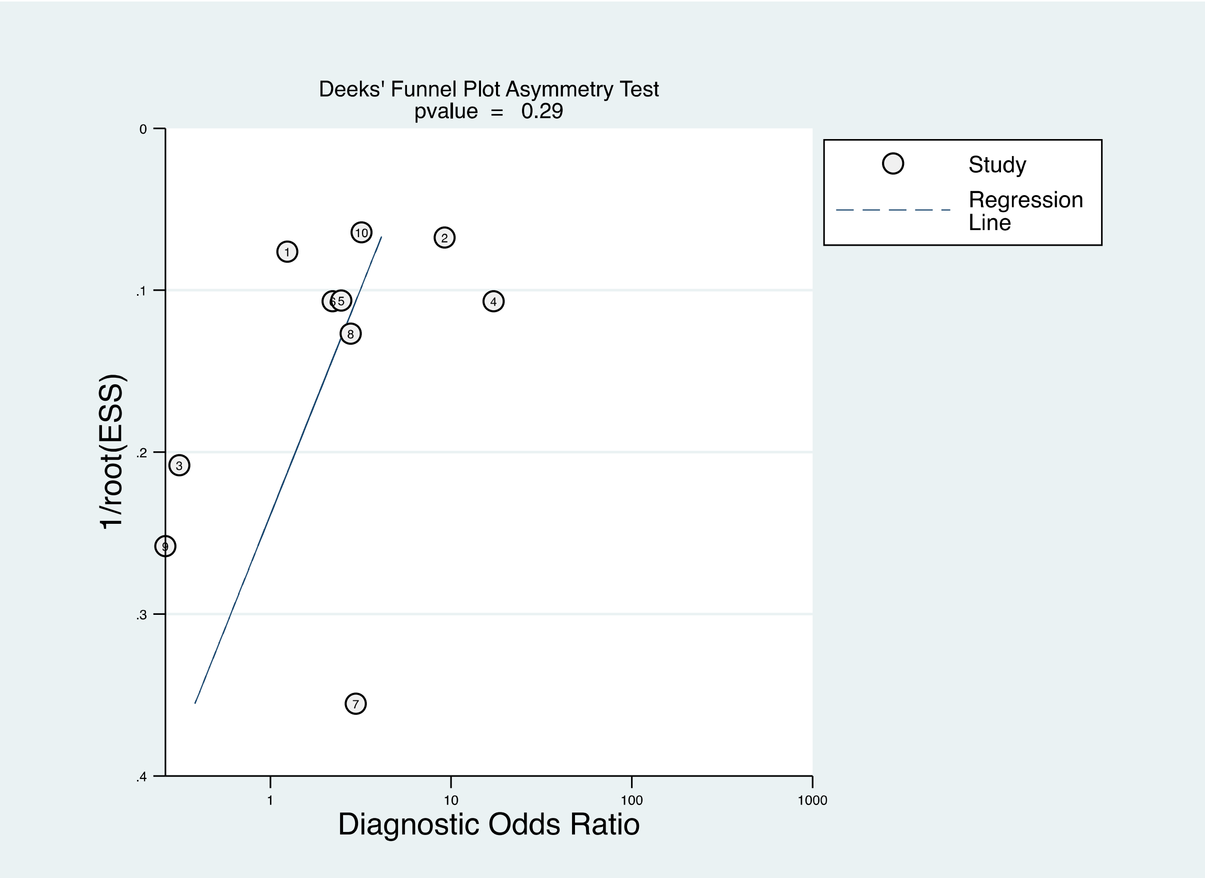

Search strategy

Syndromic STI case management systematic review:

Search methodology and search strategies

1 Search methodology ... 20
1.1 Sources searched ... 20
1.2 Information management ... 20
2 Results ... 21
2.1 Genital ulcers syndromes ... 21
3 Appendix: Search strategies ... 22
3.1 Genital ulcer syndromes ... 22
3.1.1 OvidSP MEDLINE and Epub Ahead of Print, In-Process & Other Non-Indexed Citations and Daily ... 22
3.1.2 OvidSP Embase ... 25
3.1.3 OvidSP Global Health ... 28
3.1.4 OvidSP Northern Light Live Sciences Conference Abstracts ... 31
3.1.5 Ebsco CINAHL Plus ... 33
3.1.6 Ebsco Africa-Wide Information ... 36
3.1.7 Clarivate Analytics Web of Science ... 39
3.1.8 BIREME/PAHO/WHO LILACS ... 40
5 Search methodology ... 42
6 Databases ... 42
6.1 Information management ... 43
7 Results ... 43
8 References ... 44
9 Appendix: Search strategies ... 44
9.1 Medline ... 44
9.2 Embase ... 46
9.3 Global Health ... 49
9.4 CINAHL Plus ... 52
9.5 Africa-Wide Information ... 54
9.6 Web of Science Core Collection ... 56
9.7 LILACS ... 58

1 Search methodology

After discussion with the project team, it was decided that three separate searches would be conducted: one for each of the syndromes under investigation. It was also decided that papers focusing on an analysis of the accuracy or sensitivity of the syndromic management approach would be separated from papers covering other aspects. The search for each syndrome has been constructed as below.

- Concept 1: syndromic management
- Concept 2: syndrome under investigation
- Concept 3: diagnostic accuracy and sensitivity papers
- Results group 1: concept 1 AND concept 2 AND concept 3
- Results group 2: (concept 1 AND concept 2) NOT Results group 1

A draft search strategy was compiled in the OvidSP Medline database by an experienced information specialist. The search strategy included strings of terms, synonyms and controlled vocabulary terms (where available). As the syndromic management approach was not introduced until 1996, the search was limited to papers published in 1995 or after. No other limits were added. This search strategy was refined with the project team until the results retrieved reflected the scope of the project. The agreed OvidSP Medline search was adapted for each database to incorporate database-specific syntax and controlled vocabularies. Full details of the search strings used for each database can be found in the appendix.

1.1 Sources searched

The following databases were searched on 12 and 13 September 2019.

- Ovid SP Medline and Epub Ahead of Print, In-Process & Other Non-Indexed Citations and Daily, 1946 to September 11, 2019
- OvidSP Embase, 1974 to 11 September 2019
- OvidSP Global Health, 1910 to week 35, 2019
- OvidSP Northern Light Life Sciences Conference Abstracts, 2010 to Week 34, 2019
- Ebsco CINAHL Plus, complete database
- Ebsco Africa-Wide Information, complete database
- Clarivate Analytics Web of Science Core Collection, consisting of the following databases:
    - Science Citation Index Expanded (SCI-EXPANDED), 1970 - present
    - Social Sciences Citation Index (SSCI), 1970 - present
    - Arts & Humanities Citation Index (A&HCI), 1975 - present
    - Conference Proceedings Citation Index - Science (CPCI-S), 1990 - present
    - Conference Proceedings Citation Index - Social Science & Humanities (CPCI-SSH), 1990 - present
    - Emerging Sources Citation Index (ESCI), 2015 – present
- Data last updated on 11 September 2019
- BIREME/PAHO/WHO Virtual Health Library LILACS, complete database

1.2 Information management

All citations identified by our searches were imported into EndNote X9 software. Duplicates were identified and removed using the method described on the LAS blog.[1]

2 Results

2.1 Genital ulcers syndromes

A total of 14,190- results were retrieved by the search. 4286 (30%) were identified as duplicates. Number of results pre-and post-deduplication are listed in the table below.

Database name	Diagnostic accuracy: Total number of results	Diagnostic accuracy: Number of results once duplicates removed	Other papers: Total number of results	Other papers: Number of results once duplicates removed
Ovid SP Medline and Epub Ahead of Print, In-Process & Other Non-Indexed Citations and Daily	1753	1748	941	940
OvidSP Embase	4687	3564	2590	2063
OvidSP Global Health	1159	428	398	202
OvidSP Northern Light Life Sciences Conference Abstracts	60	30	74	39
Ebsco CINAHL Plus	526	120	470	209
Ebsco Africa-Wide Information	287	26	63	13

[1] Falconer, Jane, Removing duplicates from an EndNote library. Library & Archives Service Blog: London School of Hygiene & Tropical Medicine. 2018. [online blog] http://blogs.lshtm.ac.uk/library/2018/12/07/removing-duplicates-from-an-endnote-library/.

Clarivate Analytics Web of Science Core Collection	893	346	216	108
BIREME/PAHO/WHO Virtual Health Library LILACS	44	41	29	27
**Total**	**9409**	**6303**	**4781**	**3601**

3 Appendix: Search strategies

This appendix provides full details of all search strings used for bibliographic databases, with dates and number of references returned and notes explaining any unusual search techniques or syntax. The EndNote X9 import order is provided, as the deduplication technique keeps the first uploaded copy of the reference by default.

In all searches, numbers in parentheses at the end of each row show the number of hits retrieved.

3.1 Genital ulcer syndromes

3.1.1 OvidSP MEDLINE and Epub Ahead of Print, In-Process & Other Non-Indexed Citations and Daily

Database name	Medline and Epub Ahead of Print, In-Process & Other Non-Indexed Citations and Daily
Database platform	OvidSP
Dates of database coverage	1946 to September 12, 2019
Date searched	13 September 2019
Searched by	JF
Number of results	Diagnostic accuracy: 1753<br>Others: 940

EndNote import order	1 (Medline) and 3 (Medline in process)
Number of results once duplicates removed	Diagnostic accuracy: 1748<br>Others: 149
Search strategy notes	Search lines ending in a '/' are subject heading searches.<br>Search lines beginning 'exp' are exploded subject heading searches.<br>Search lines ending in .ti,ab. search in the title and abstract only.<br>Search lines ending in .ab. search in the abstract only<br>or/*x*-*y* combines search sets in the range *x*-*y* with Boolean operator OR.<br>* is used for truncation of words.<br>? is used for optional wildcards.<br># is used for compulsory wildcards.<br>adj*n* searches for words within *n* words of each other.

1 ((ulcer* or vesicle* or papule* or chancr* or granulom* or sore* or lesion* or pustule?) adj3 (genital* or vener* or penile or penis or anogenital or vagin*)).ti,ab. (6625)
2 gud.ti,ab. (240)
3 Herpes Simplex/ (14065)
4 Herpes genitalis/ (4595)
5 simplexvirus/ (17144)
6 "herpesvirus 1, human"/ (10099)
7 "herpesvirus 2, human"/ (4216)
8 herpes genitalis.ti,ab. (333)
9 genital herpes.ti,ab. (2997)
10 herpes virus.ti,ab. (5384)
11 herpes simplex.ti,ab. (39410)
12 herpesvirus.ti,ab. (21627)
13 simplexvirus.ti,ab. (30)
14 "HSV-1".ti,ab. (11806)
15 "HHV-1".ti,ab. (77)
16 "HSV-2".ti,ab. (5953)
17 "HHV-2".ti,ab. (36)
18 exp Trichomonas Infections/ (6051)
19 Trichomonas vaginalis/ (3497)
20 trichomoniasis.ti,ab. (3003)
21 trichomoniases.ti,ab. (2)
22 trichomonas vaginalis.ti,ab. (4532)

23 trichomonas vaginitides.ti,ab. (0)
24 trichomonas vaginitis.ti,ab. (253)
25 trichomonas vaginali.ti,ab. (2)
26 exp Syphilis/ (27234)
27 Treponema pallidum/ (3921)
28 syphilis.ti,ab. (24139)
29 chancre.ti,ab. (461)
30 treponema pallidum.ti,ab. (4082)
31 condylomata lata.ti,ab. (46)
32 Granuloma Inguinale/ (589)
33 Calymmatobacterium/ (71)
34 granuloma inguinale.ti,ab. (356)
35 Calymmatobacterium granulomatis.ti,ab. (37)
36 granuloma venereum.ti,ab. (66)
37 donovanosis.ti,ab. (231)
38 Chlamydia/ (2875)
39 chlamydia trachomatis/ (11716)
40 chlamydia.ti,ab. (24181)
41 lymphogranuloma venereum/ (1564)
42 lymphogranuloma venereum.ti,ab. (1083)
43 LGV.ti,ab. (558)
44 lymphogranuloma inguinale.ti,ab. (81)
45 Chancroid/ (858)
46 Haemophilus ducreyi/ (650)
47 chancroid?.ti,ab. (880)
48 haemophilus ducreyi.ti,ab. (751)
49 hemophilus ducreyi.ti,ab. (37)
50 Gonorrhea/ (13550)
51 Neisseria gonorrhoeae/ (9524)
52 gonorrhea.ti,ab. (6662)
53 neisseria gonorrhoeae.ti,ab. (9392)
54 gonococcal urethritis.ti,ab. (1241)
55 gonococci.ti,ab. (1988)
56 or/1-55 [GENITAL ULCER DISEASE] (162973)
57 Syndrome/ (114541)
58 algorithms/ (241132)
59 Practice Guidelines as Topic/ (111972)
60 Risk Assessment/ (247541)
61 Critical Pathways/ (6388)
62 flowchart?.ti,ab. (1157)
63 flow-chart?.ti,ab. (1400)
64 algorithm?.ti,ab. (227742)
65 clinical pathway?.ti,ab. (3211)
66 risk assessment?.ti,ab. (58804)
67 syndromically.ti,ab. (42)
68 syndromic.ti,ab. (10684)
69 decision tree.ti,ab. (6435)

70 detection tool?.ti,ab. (1067)
71 or/57-70 [SYNDROMIC MANAGEMENT] (877723)
72 exp "Sensitivity and Specificity"/ (560793)
73 effective*.ti,ab. (1834458)
74 sensitiv*.ti,ab. (1319730)
75 specific*.ti,ab. (2961344)
76 perfor*.ti,ab. (3119029)
77 predict*.ti,ab. (1471621)
78 quantitative.ti,ab. (578506)
79 random*.ti,ab. (1071982)
80 predictive value?.ti,ab. (101956)
81 study.ab. (6617822)
82 efficacy.ti,ab. (750800)
83 (accuracy or accurate).ti,ab. (640473)
84 or/72-83 [SENSITIVITY AND SPECIFICITY] (12667961)
85 56 and 71 (3132)
86 84 and 85 (1843)
87 85 not 86 (1289)
88 limit 86 to yr="1995 -Current" (1753)
89 limit 87 to yr="1995 -Current" (941)

3.1.2 OvidSP Embase

Database name	Embase
Database platform	OvidSP
Dates of database coverage	1974 to September 12, 2019
Date searched	13 September 2019
Searched by	JF
Number of results	Diagnostic accuracy: 4687<br>Others: 2590
EndNote import order	2

Number of results once duplicates removed	Diagnostic accuracy: 3564<br>Others: 2063
Search strategy notes	Search lines ending in a '/' are subject heading searches.<br>Search lines beginning 'exp' are exploded subject heading searches.<br>Search lines ending in .ti,ab. search in the title and abstract only.<br>Search lines ending in .ab. search in the abstract only<br>or/*x-y* combines search sets in the range *x-y* with Boolean operator OR.<br>* is used for truncation of words.<br>? is used for optional wildcards.<br># is used for compulsory wildcards.<br>adj*n* searches for words within *n* words of each other.

1 syndrome/ (48783)
2 algorithm/ (248599)
3 practice guideline/ (392504)
4 risk assessment/ (530005)
5 clinical pathway/ (8175)
6 "decision tree"/ (11597)
7 flowchart?.ti,ab. (1832)
8 flow-chart?.ti,ab. (2329)
9 algorithm?.ti,ab. (286930)
10 clinical pathway?.ti,ab. (5266)
11 risk assessment?.ti,ab. (79550)
12 syndromically.ti,ab. (66)
13 syndromic.ti,ab. (14440)
14 decision tree.ti,ab. (9792)
15 detection tool?.ti,ab. (1369)
16 or/1-15 [SYNDROMIC MANAGEMENT] (1358607)
17 "sensitivity and specificity"/ (337091)
18 clinical effectiveness/ (117320)
19 predictive value/ (156255)
20 diagnostic accuracy/ (242975)
21 effective*.ti,ab. (2412051)
22 sensitiv*.ti,ab. (1647888)
23 specific*.ti,ab. (3721765)
24 perfor*.ti,ab. (4358098)
25 predict*.ti,ab. (1981275)
26 quantitative.ti,ab. (717461)
27 random*.ti,ab. (1456620)
28 predictive value?.ti,ab. (152771)
29 study.ab. (8906138)

30 efficacy.ti,ab. (1105540)
31 (accuracy or accurate).ti,ab. (816693)
32 or/17-31 [SENSITIVITY AND SPECIFICITY] (16284740)
33 genital ulcer/ (3817)
34 ((ulcer* or vesicle* or papule* or chancr* or granulom* or sore* or lesion* or pustule?) adj3 (genital* or vener* or penile or penis or anogenital or vagin*)).ti,ab. (8895)
35 gud.ti,ab. (274)
36 herpes simplex/ (18201)
37 genital herpes/ (5854)
38 herpes labialis/ (2027)
39 simplexvirus/ (778)
40 exp Herpes simplex virus/ (32971)
41 herpes genitalis.ti,ab. (417)
42 genital herpes.ti,ab. (3664)
43 herpes virus.ti,ab. (7179)
44 herpes simplex.ti,ab. (44237)
45 herpesvirus.ti,ab. (22941)
46 simplexvirus.ti,ab. (40)
47 "HSV-1".ti,ab. (13829)
48 "HHV-1".ti,ab. (101)
49 "HSV-2".ti,ab. (7049)
50 "HHV-2".ti,ab. (50)
51 exp Trichomonas/ (7455)
52 trichomoniasis.ti,ab. (2861)
53 trichomoniases.ti,ab. (3)
54 trichomonas vaginalis.ti,ab. (4915)
55 trichomonas vaginitides.ti,ab. (0)
56 trichomonas vaginitis.ti,ab. (134)
57 trichomonas vaginali.ti,ab. (0)
58 exp syphilis/ (23988)
59 Treponema pallidum/ (5721)
60 syphilis.ti,ab. (22323)
61 chancre.ti,ab. (409)
62 treponema pallidum.ti,ab. (4352)
63 condylomata lata.ti,ab. (57)
64 granuloma inguinale/ (577)
65 klebsiella/ (15548)
66 klebsiella granulomatis/ (26)
67 granuloma inguinale.ti,ab. (205)
68 Calymmatobacterium granulomatis.ti,ab. (36)
69 granuloma venereum.ti,ab. (20)
70 donovanosis.ti,ab. (254)
71 chlamydia/ (7116)
72 chlamydia trachomatis/ (18306)

73 chlamydia.ti,ab. (30467)
74 lymphogranuloma venereum/ (1420)
75 lymphogranuloma venereum.ti,ab. (1029)
76 LGV.ti,ab. (800)
77 lymphogranuloma inguinale.ti,ab. (14)
78 ulcus molle/ (1229)
79 haemophilus ducreyi/ (1057)
80 chancroid?.ti,ab. (855)
81 haemophilus ducreyi.ti,ab. (806)
82 hemophilus ducreyi.ti,ab. (20)
83 gonorrhea/ (16168)
84 gonococcal urethritis/ (713)
85 Neisseria gonorrhoeae/ (15157)
86 gonorrhea.ti,ab. (6760)
87 neisseria gonorrhoeae.ti,ab. (10751)
88 gonococcal urethritis.ti,ab. (1339)
89 gonococci.ti,ab. (1982)
90 or/33-89 [GENITAL ULCER SYNDROME] (200634)
91 16 and 90 (7576)
92 32 and 91 (4827)
93 remove duplicates from 92 (4770)
94 91 not 92 (2749)
95 remove duplicates from 94 (2721)
96 limit 93 to yr="1995 -Current" (4687)
97 limit 95 to yr="1995 -Current" (2590)

3.1.3 OvidSP Global Health

Database name	Global Health
Database platform	OvidSP
Dates of database coverage	1910 to 2019 week 36
Date searched	13 September 2019
Searched by	JF
Number of results	Diagnostic accuracy: 1159<br>Others: 398

EndNote import order	4
Number of results once duplicates removed	Diagnostic accuracy: 428<br>Others: 202
Search strategy notes	Search lines ending in a '/' are subject heading searches.<br>Search lines beginning 'exp' are exploded subject heading searches.<br>Search lines ending in .ti,ab. search in the title and abstract only.<br>Search lines ending in .ab. search in the abstract only<br>or/*x-y* combines search sets in the range *x-y* with Boolean operator OR.<br>* is used for truncation of words.<br>? is used for optional wildcards.<br># is used for compulsory wildcards.<br>adj*n* searches for words within *n* words of each other.

1 syndromic management/ (14)
2 algorithms/ (4855)
3 guidelines/ (44828)
4 risk assessment/ (50405)
5 flowchart?.ti,ab. (179)
6 flow-chart?.ti,ab. (285)
7 algorithm?.ti,ab. (13492)
8 clinical pathway?.ti,ab. (253)
9 risk assessment?.ti,ab. (21519)
10 syndromically.ti,ab. (22)
11 syndromic.ti,ab. (1677)
12 decision tree.ti,ab. (1055)
13 detection tool?.ti,ab. (198)
14 or/1-13 [SYNDROMIC MANAGEMENT] (115032)
15 sensitivity analysis/ (1152)
16 program effectiveness/ (56)
17 diagnostic value/ (3864)
18 effective*.ti,ab. (343639)
19 sensitiv*.ti,ab. (211270)
20 specific*.ti,ab. (417079)
21 perfor*.ti,ab. (390692)
22 predict*.ti,ab. (176468)
23 quantitative.ti,ab. (84606)

24 random*.ti,ab. (210419)
25 predictive value?.ti,ab. (15284)
26 study.ab. (1311320)
27 efficacy.ti,ab. (123272)
28 (accuracy or accurate).ti,ab. (73687)
29 or/15-28 [SENSITIVITY AND SPECIFICITY] (2112109)
30 genital ulcers/ (689)
31 ((ulcer* or vesicle* or papule* or chancr* or granulom* or sore* or lesion* or pustule?) adj3 (genital* or vener*or penile or penis or anogenital or vagin*)).ti,ab. (2171)
32 gud.ti,ab. (137)
33 exp neisseria gonorrhoeae/ (10030)
34 gonorrhea.ti,ab. (2248)
35 neisseria gonorrhoeae.ti,ab. (4513)
36 gonococcal urethritis.ti,ab. (945)
37 gonococci.ti,ab. (1655)
38 chlamydia/ (11893)
39 chlamydia trachomatis/ (9209)
40 chlamydia.ti,ab. (10974)
41 lymphogranuloma venereum.ti,ab. (833)
42 LGV.ti,ab. (297)
43 lymphogranuloma inguinale.ti,ab. (375)
44 exp herpes simplex/ (6816)
45 simplexvirus/ (5289)
46 human herpesvirus 1/ (2793)
47 human herpesvirus 2/ (2816)
48 herpes genitalis.ti,ab. (150)
49 genital herpes.ti,ab. (1406)
50 herpes virus.ti,ab. (1778)
51 herpes simplex.ti,ab. (10032)
52 herpesvirus.ti,ab. (4918)
53 simplexvirus.ti,ab. (10)
54 "HSV-1".ti,ab. (2486)
55 "HHV-1".ti,ab. (45)
56 "HSV-2".ti,ab. (2463)
57 "HHV-2".ti,ab. (19)
58 exp treponema pallidum/ (17403)
59 syphilis.ti,ab. (16714)
60 chancre.ti,ab. (522)
61 treponema pallidum.ti,ab. (2594)
62 condylomata lata.ti,ab. (14)
63 Trichomonas/ (6758)
64 Trichomonas vaginalis/ (4930)
65 trichomoniasis.ti,ab. (2245)
66 trichomoniases.ti,ab. (2)
67 trichomonas vaginalis.ti,ab. (4559)
68 trichomonas vaginitides.ti,ab. (0)
69 trichomonas vaginitis.ti,ab. (127)

70 trichomonas vaginali.ti,ab. (0)
71 exp klebsiella granulomatis/ (568)
72 granuloma inguinale.ti,ab. (427)
73 Calymmatobacterium granulomatis.ti,ab. (27)
74 granuloma venereum.ti,ab. (180)
75 donovanosis.ti,ab. (119)
76 exp haemophilus ducreyi/ (812)
77 chancroid?.ti,ab. (685)
78 haemophilus ducreyi.ti,ab. (331)
79 hemophilus ducreyi.ti,ab. (11)
80 or/30-79 [GENITAL ULCER DISEASE] (61706)
81 14 and 80 (1730)
82 29 and 81 (1251)
83 81 not 82 (479)
84 limit 82 to yr="1995-Current" (1159)
85 limit 83 to yr="1995-Current" (398)

3.1.4 OvidSP Northern Light Live Sciences Conference Abstracts

Database name	Northern Light Life Sciences Conference Abstracts
Database platform	OvidSP
Dates of database coverage	2010 to 2019 week 34
Date searched	13 September 2019
Searched by	JF
Number of results	Diagnostic accuracy: 60<br>Others: 74
EndNote import order	9
Number of results once duplicates removed	Diagnostic accuracy: 30<br>Others: 39

Search strategy notes	Search lines ending in .ti,ab. search in the title and abstract only.<br>Search lines ending in .ab. search in the abstract only<br>or/*x-y* combines search sets in the range *x-y* with Boolean operator OR.<br>* is used for truncation of words.<br>? is used for optional wildcards.<br># is used for compulsory wildcards.<br>adj*n* searches for words within *n* words of each other.

1 ((ulcer* or vesicle* or papule* or chancr* or granulom* or sore* or lesion* or pustule?) adj3 (genital* or vener* or penile or penis or anogenital or vagin*)).ti,ab. (251)
2 gud.ti,ab. (11)
3 Herpes Simplex/ (3331)
4 Herpes genitalis/ (226)
5 herpes genitalis.ti,ab. (3)
6 genital herpes.ti,ab. (67)
7 herpes virus.ti,ab. (305)
8 herpes simplex.ti,ab. (1254)
9 herpesvirus.ti,ab. (534)
10 simplexvirus.ti,ab. (0)
11 "HSV-1".ti,ab. (734)
12 "HHV-1".ti,ab. (3)
13 "HSV-2".ti,ab. (349)
14 "HHV-2".ti,ab. (1)
15 exp Trichomonas Infections/ (13)
16 trichomoniasis.ti,ab. (58)
17 trichomoniases.ti,ab. (0)
18 trichomonas vaginalis.ti,ab. (176)
19 trichomonas vaginitides.ti,ab. (0)
20 trichomonas vaginitis.ti,ab. (1)
21 trichomonas vaginali.ti,ab. (0)
22 exp Syphilis/ (3744)
23 syphilis.ti,ab. (1475)
24 chancre.ti,ab. (14)
25 treponema pallidum.ti,ab. (103)
26 condylomata lata.ti,ab. (3)
27 Granuloma Inguinale/ (14)
28 granuloma inguinale.ti,ab. (1)
29 Calymmatobacterium granulomatis.ti,ab. (0)
30 granuloma venereum.ti,ab. (2)
31 donovanosis.ti,ab. (4)
32 chlamydia.ti,ab. (1401)
33 lymphogranuloma venereum/ (108)
34 lymphogranuloma venereum.ti,ab. (47)
35 LGV.ti,ab. (65)

36 lymphogranuloma inguinale.ti,ab. (0)
37 Chancroid/ (27)
38 chancroid?.ti,ab. (6)
39 haemophilus ducreyi.ti,ab. (17)
40 hemophilus ducreyi.ti,ab. (0)
41 Gonorrhea/ (1433)
42 gonorrhea.ti,ab. (408)
43 neisseria gonorrhoeae.ti,ab. (436)
44 gonococcal urethritis.ti,ab. (21)
45 gonococci.ti,ab. (17)
46 or/1-45 [GENITAL ULCER DISEASE] (10568)
47 flowchart?.ti,ab. (192)
48 flow-chart?.ti,ab. (231)
49 algorithm?.ti,ab. (22310)
50 clinical pathway?.ti,ab. (587)
51 risk assessment?.ti,ab. (6324)
52 syndromically.ti,ab. (0)
53 syndromic.ti,ab. (1553)
54 decision tree.ti,ab. (444)
55 detection tool?.ti,ab. (108)
56 or/47-55 [SYNDROMIC MANAGEMENT] (31504)
57 effective*.ti,ab. (186775)
58 sensitiv*.ti,ab. (93744)
59 specific*.ti,ab. (195961)
60 perfor*.ti,ab. (168177)
61 predict*.ti,ab. (206332)
62 quantitative.ti,ab. (33674)
63 random*.ti,ab. (69286)
64 predictive value?.ti,ab. (8803)
65 study.ab. (458030)
66 efficacy.ti,ab. (90744)
67 (accuracy or accurate).ti,ab. (65701)
68 or/57-67 [SENSITIVITY AND SPECIFICITY] (1226243)
69 46 and 56 (134)
70 68 and 69 (60)
71 69 not 70 (74)

3.1.5 Ebsco CINAHL Plus

Database name	CINAHL Plus
Database platform	Ebsco

Dates of database coverage	Complete database
Date searched	13 September 2019
Searched by	JF
Number of results	Diagnostic accuracy: 526<br>Others: 470
EndNote import order	5
Number of results once duplicates removed	Diagnostic accuracy: 120<br>Others: 209
Search strategy notes	* is used for truncation.<br>N/*n* is used to find words within *n* words of each other.<br>Searches beginning MH are subject heading searches.<br>Subject heading searches ending with + are exploded.<br>Searches beginning TI search the title only.<br>Searches beginning AB search the abstract only.

S1 (TI (ulcer* or vesicle* or papule* or chancr* or granulom* or sore* or lesion* or pustule#) N3 (genital* or vener* or penile or penis or anogenital or vagin*)) OR (AB (ulcer* or vesicle* or papule* or chancr* or granulom* or sore* or lesion* or pustule#) N3 (genital* or vener* or penile or penis or anogenital or vagin*)) (1,050)
S2 (TI gud) OR (AB gud) (49)
S3 (MH "Herpes Simplex") (2,024)
S4 (MH "Herpes Genitalis") (1,260)
S5 (MH "Herpesviruses") (2,979)
S6 (TI "herpes genitalis") OR (AB "herpes genitalis") (28)
S7 (TI "genital herpes") OR (AB "genital herpes") (627)
S8 (TI "herpes virus") OR (AB "herpes virus") (356)
S9 (TI "herpes simplex") OR (AB "herpes simplex") (2,877)
S10 (TI herpesvirus) OR (AB herpesvirus) (855)
S11 (TI simplexvirus) OR (AB simplexvirus) (0)
S12 (TI "HSV-1") OR (AB "HSV-1") (448)
S13 (TI "HHV-1") OR (AB "HHV-1") (6)
S14 (TI "HSV-2") OR (AB "HSV-2") (648)
S15 (TI "HHV-2") OR (AB "HHV-2") (2)
S16 (MH "Syphilis+") (3,543)

S17 (TI syphilis) OR (AB syphilis) (3,683)
S18 (TI chancre) OR (AB chancre) (38)
S19 (TI "treponema pallidum") OR (AB "treponema pallidum") (435)
S20 (TI "condylomata lata") OR (AB "condylomata lata") (3)
S21 (MH "Chlamydia Infections") (3,701)
S22 (MH "Lymphogranuloma Venereum") (213)
S23 (MH "Chlamydia+") (1,894)
S24 (TI chlamydia) OR (AB chlamydia) (4,338)
S25 (TI "lymphogranuloma venereum") OR (AB "lymphogranuloma venereum") (164)
S26 (TI LGV) OR (AB LGV) (75)
S27 (TI "lymphogranuloma inguinale") OR (AB "lymphogranuloma inguinale") (0)
S28 (MH "Gonorrhea") (2,657)
S29 (MH "Neisseria") (1,056)
S30 (TI gonorrhea) OR (AB gonorrhea) (1,976)
S31 (TI "neisseria gonorrhoeae") OR (AB "neisseria gonorrhoeae") (1,228)
S32 (TI "gonococcal urethritis") OR (AB "gonococcal urethritis") (82)
S33 (TI gonococci) OR (AB gonococci) (74)
S34 (MH "Trichomonas Vaginitis") (457)
S35 (TI trichomoniasis) OR (AB trichomoniasis) (410)
S36 (TI trichomoniases) OR (AB trichomoniases) (1)
S37 (TI "trichomonas vaginalis") OR (AB "trichomonas vaginalis") (601)
S38 (TI "trichomonas vaginali") OR (AB "trichomonas vaginali") (0)
S39 (MH "Granuloma Inguinale") (32)
S40 (MH "Klebsiella") (1,403)
S41 (TI "granuloma inguinale") OR (AB "granuloma inguinale") (13)
S42 (TI "Calymmatobacterium granulomatis") OR (AB "Calymmatobacterium granulomatis") (5)
S43 (TI "granuloma venereum") OR (AB "granuloma venereum") (0)
S44 (TI donovanosis) OR (AB donovanosis) (23)
S45 (MH "Chancroid") (31)
S46 (TI chancroid*) OR (AB chancroid*) (68)
S47 (TI "haemophilus ducreyi") OR (AB "haemophilus ducreyi") (62)
S48 (TI "hemophilus ducreyi") OR (AB "hemophilus ducreyi") (1)
S49 S1 or S2 or S3 or S4 or S5 or S6 or S7 or S8 or S9 or S10 or S11 or S12 or S13 or S14 or S15 or S16 or S17 or S18 or S19 or S20 or S21 or S22 or S23 or S24 or S25 or S26 or S27 or S28 or S29 or S30 or S31 or S32 or S33 or S34 or S35 or S36 or S37 or S38 or S39 or S40 or S41 or S42 or S43 or S44 or S45 or S46 or S47 or S48 (20,872)
S50 (MH "Syndrome") (9,946)
S51 (MH "Algorithms") (32,643)
S52 (MH "Decision Trees") (1,983)
S53 (MH "Practice Guidelines") (69,181)
S54 (MH "Risk Assessment") (84,602)
S55 (MH "Critical Path") (4,864)
S56 (TI flowchart#) OR (AB flowchart#) (424)
S57 (TI "flow-chart#") OR (AB "flow-chart#") (332)

S58 (TI algorithm#) OR (AB algorithm#) (28,846)
S59 (TI "clinical pathway#") OR (AB "clinical pathway#") (1,716)
S60 (TI "risk assessment#") OR (AB "risk assessment#") (13,724)
S61 (TI syndromically) OR (AB syndromically) (11)
S62 (TI syndromic) OR (AB syndromic) (1,958)
S63 (TI "decision tree") OR (AB "decision tree") (1,547)
S64 (TI "detection tool#") OR (AB "detection tool#") (156)
S65 S50 OR S51 OR S52 OR S53 OR S54 OR S55 OR S56 OR S57 OR S58 OR S59 OR S60 OR S61 OR S62 OR S63 OR S64 (220,725)
S66 (MH "Sensitivity and Specificity") (75,082)
S67 (MH "Predictive Value of Tests") (45,113)
S68 (TI effective*) OR (AB effective*) (381,825)
S69 (TI sensitiv*) OR (AB sensitiv*) (136,607)
S70 (TI specific*) OR (AB specific*) (348,685)
S71 (TI perfor*) or (AB perfor*) (510,687)
S72 (TI predict*) OR (AB predict*) (307,915)
S73 (TI quantitative) OR (AB quantitative) (69,877)
S74 (TI random*) OR (AB random*) (300,173)
S75 (TI "predictive value#") OR (AB "predictive value#") (22,469)
S76 (AB study) (1,508,670)
S77 (TI efficacy) OR (AB efficacy) (160,771)
S78 (TI (accuracy or accurate)) OR (AB (accuracy or accurate)) (101,062)
S79 S66 OR S67 OR S68 OR S69 OR S70 OR S71 OR S72 OR S73 OR S74 OR S75 OR S76 OR S77 OR S78 (2,217,113)
S80 S49 AND S65 (1,009)
S81 S79 AND S80 (529)
S82 S80 NOT S81 (480)
S83 S81
Limiters - Published Date: 19950101-20201231 (526)
S84 S82
Limiters - Published Date: 19950101-20201231 (470)

3.1.6 Ebsco Africa-Wide Information

Database name	Africa-Wide Information
Database platform	Ebsco
Dates of database coverage	Complete database
Date searched	13 September 2019

Searched by	JF
Number of results	Diagnostic accuracy: 287<br>Others: 63
EndNote import order	6
Number of results once duplicates removed	Diagnostic accuracy: 26<br>Others: 13
Search strategy notes	* is used for truncation.<br>N/*n* is used to find words within *n* words of each other.<br>Searches beginning TI search the title only.<br>Searches beginning AB search the abstract only.

S1 (TI (ulcer* or vesicle* or papule* or chancr* or granulom* or sore* or lesion* or pustule#) N3 (genital* or vener* or penile or penis or anogenital or vagin*)) OR (AB (ulcer* or vesicle* or papule* or chancr* or granulom* or sore* or lesion* or pustule#) N3 (genital* or vener* or penile or penis or anogenital or vagin*)) (1,261)
S2 (TI gud) OR (AB gud) (134)
S3 (TI "herpes genitalis") OR (AB "herpes genitalis") (27)
S4 (TI "genital herpes") OR (AB "genital herpes") (290)
S5 (TI "herpes virus") OR (AB "herpes virus") (377)
S6 (TI "herpes simplex") OR (AB "herpes simplex") (1,825)
S7 (TI herpesvirus) OR (AB herpesvirus) (1,195)
S8 (TI simplexvirus) OR (AB simplexvirus) (1)
S9 (TI "HSV-1") OR (AB "HSV-1") (344)
S10 (TI "HHV-1") OR (AB "HHV-1") (5)
S11 (TI "HSV-2") OR (AB "HSV-2") (618)
S12 (TI "HHV-2") OR (AB "HHV-2") (4)
S13 (TI syphilis) OR (AB syphilis) (3,168)
S14 (TI chancre) OR (AB chancre) (102)
S15 (TI "treponema pallidum") OR (AB "treponema pallidum") (435)
S16 (TI "condylomata lata") OR (AB "condylomata lata") (2)
S17 (TI chlamydia) OR (AB chlamydia) (1,986)
S18 (TI "lymphogranuloma venereum") OR (AB "lymphogranuloma venereum") (110)
S19 (TI LGV) OR (AB LGV) (43)
S20 (TI "lymphogranuloma inguinale") OR (AB "lymphogranuloma inguinale") (3)
S21 (TI gonorrhea) OR (AB gonorrhea) (780)

S22 (TI "neisseria gonorrhoeae") OR (AB "neisseria gonorrhoeae") (927)
S23 (TI "gonococcal urethritis") OR (AB "gonococcal urethritis") (105)
S24 (TI gonococci) OR (AB gonococci) (96)
S25 (TI trichomoniasis) OR (AB trichomoniasis) (444)
S26 (TI trichomoniases) OR (AB trichomoniases) (3)
S27 (TI "trichomonas vaginalis") OR (AB "trichomonas vaginalis") (638)
S28 (TI "trichomonas vaginali") OR (AB "trichomonas vaginali") (0)
S29 (TI "granuloma inguinale") OR (AB "granuloma inguinale") (62)
S30 (TI "Calymmatobacterium granulomatis") OR (AB "Calymmatobacterium granulomatis") (14)
S31 (TI "granuloma venereum") OR (AB "granuloma venereum") (4)
S32 (TI donovanosis) OR (AB donovanosis) (53)
S33 (TI chancroid*) OR (AB chancroid*) (259)
S34 (TI "haemophilus ducreyi") OR (AB "haemophilus ducreyi") (168)
S35 (TI "hemophilus ducreyi") OR (AB "hemophilus ducreyi") (11)
S36 S1 or S2 or S3 or S4 or S5 or S6 or S7 or S8 or S9 or S10 or S11 or S12 or S13 or S14 or S15 or S16 or S17 or S18 or S19 or S20 or S21 or S22 or S23 or S24 or S25 or S26 or S27 or S28 or S29 or S30 or S31 or S32 or S33 or S34 or S35 (10,551)
S37 (TI flowchart#) OR (AB flowchart#) (107)
S38 (TI "flow-chart#") OR (AB "flow-chart#") (255)
S39 (TI algorithm#) OR (AB algorithm#) (7,541)
S40 (TI "clinical pathway#") OR (AB "clinical pathway#") (73)
S41 (TI "risk assessment#") OR (AB "risk assessment#") (3,147)
S42 (TI syndromically) OR (AB syndromically) (18)
S43 (TI syndromic) OR (AB syndromic) (853)
S44 (TI "decision tree") OR (AB "decision tree") (296)
S45 (TI "detection tool#") OR (AB "detection tool#") (66)
S46 S37 OR S38 OR S39 OR S40 OR S41 OR S42 OR S43 OR S44 OR S45 (12,111)
S47 (TI effective*) OR (AB effective*) (130,444)
S48 (TI sensitiv*) OR (AB sensitiv*) (58,628)
S49 (TI specific*) OR (AB specific*) (161,163)
S50 (TI perfor*) or (AB perfor*) (158,452)
S51 (TI predict*) OR (AB predict*) (69,912)
S52 (TI quantitative) OR (AB quantitative) (25,475)
S53 (TI random*) OR (AB random*) (58,184)
S54 (TI "predictive value#") OR (AB "predictive value#") (5,207)
S55 (AB study) (424,315)
S56 (TI efficacy) OR (AB efficacy) (36,803)
S57 (TI (accuracy or accurate)) OR (AB (accuracy or accurate)) (28,366)
S58 S47 OR S48 OR S49 OR S50 OR S51 OR S52 OR S53 OR S54 OR S55 OR S56 OR S57 (763,177)
S59 S36 AND S46 (350)
S60 S58 AND S59 (287)
S61 S59 NOT S60 (63)
S62 S60 (287)
S63 S61 (63)

3.1.7 Clarivate Analytics Web of Science

Database name	Web of Science Core Collection:<br>• Science Citation Index Expanded (SCI-EXPANDED)<br>• Social Sciences Citation Index (SSCI)<br>• Arts & Humanities Citation Index (A&HCI)<br>• Conference Proceedings Citation Index - Science (CPCI-S)<br>• Conference Proceedings Citation Index - Social Science & Humanities (CPCI-SSH)<br>• Emerging Sources Citation Index (ESCI)
Database platform	Clarivate Analytics
Dates of database coverage	SCI-EXPANDED: 1970-present<br>SSCI: 1970-present<br>A&HCI: 1975-present<br>CPCI-S: 1990-present<br>CPCI-SSH: 1990-present<br>ESCI: 2015-present<br>Data last updated 2019-09-11
Date searched	13 September 2019
Searched by	JF
Number of results	Diagnostic accuracy: 893<br>Others: 346
EndNote import order	7
Number of results once duplicates removed	Diagnostic accuracy: 216<br>Others: 108

Search strategy notes	* is used for truncation.<br>N/*n* is used to find words within *n* words of each other.<br>Searches beginning TI search the title only.<br>Searches beginning AB search the abstract only.

All searches run on Indexes=SCI-EXPANDED, SSCI, A&HCI, CPCI-S, CPCI-SSH, ESCI Timespan=1995-2019

#1 TOPIC: (flowchart$ or flow-chart$ or algorithm$ or "clinical pathway$" or "risk assessment$" or syndromically or syndromic or "decision tree" or "detection tool$") (1,928,828)
#2 TOPIC: (effective* or sensitiv* or specific* or perfor* or predict* or quantitative or random* or "predictive value$" or efficacy or accuracy or accurate) (16,718,802)
#3 TOPIC: (gud or "herpes genitalis" or "genital herpes" or "herpes virus" or "herpes simplex" or herpesvirus or simplexvirus or "hsv-1" or "hhv-1" or "hsv-2" or "hhv-2" or syphilis or chancre or "treponema pallidum" or "condylomata lata" or chlamydia or "lymphogranuloma venereum" or lgv or "lymphogranuloma inguinale" or gonorrhea or "neisseria gonorrhoea" or "gonococcal urethritis" or gonococci or trichomoniasis or trichomoniases or "trichomonas vaginalis" or "trichomonas vaginitides" or "trichomonas vaginitis" or "trichomonas vaginali" or "granuloma inguinale" or "calymmatobacterium granulomatis" or "granuloma venereum" or donovanosis or chancroid$ or "haemophilus ducreyi" or "hemophilus ducreyi") (98,073)
#4 TOPIC: (((ulcer* or vesicle* or papule* or chancr* or granulom* or sore* or lesion* or pustule$) NEAR/3 genital*) or ((ulcer* or vesicle* or papule* or chancr* or granulom* or sore* or lesion* or pustule$) NEAR/3 vener*) or ((ulcer* or vesicle* or papule* or chancr* or granulom* or sore* or lesion* or pustule$) NEAR/3 penile) or ((ulcer* or vesicle* or papule* or chancr* or granulom* or sore* or lesion* or pustule$) NEAR/3 penis) or ((ulcer* or vesicle* or papule* or chancr* or granulom* or sore* or lesion* or pustule$) NEAR/3 anogenital) or ((ulcer* or vesicle* or papule* or chancr* or granulom* or sore* or lesion* or pustule$) NEAR/3 vagin*)) (5,113)
#5 #3 OR #4 (101,761)
#6 #1 AND #5 (1,239)
#7 #2 AND #6 (893)
#8 #6 NOT #7 (346)

3.1.8 BIREME/PAHO/WHO LILACS

Database name	LILACS
Database platform	BIREME/PAHO/WHO
Dates of database coverage	Complete database
Date searched	13 September 2019

Searched by	JF
Number of results	Diagnostic accuracy: 44<br>Others: 29
EndNote import order	8
Number of results once duplicates removed	Diagnostic accuracy: 41<br>Others: 27
Search strategy notes	$ is used for truncation.

All searches in field: words

(flowchart$ OR "flow-chart$" OR algorithm$ OR "clinical pathway$" OR "risk assessment$" OR syndromically OR syndromic OR "decision tree" OR "detection tool$") AND)effective$ OR sensitiv$ OR specific$ OR perfor$ OR predict$ OR quantitative OR random$ OR "predictive value$" OR efficacy OR accuracy OR accurate) AND (((ulcer$ OR vesicle$ or papule$ OR chancr$ OR granulom$ OR sore$ OR lesion$ OR pustule$) AND (genital$ OR vener$ OR penile OR penis OR anogenital OR vagin$)) OR "herpes genitalis" OR "herpes virus" OR "herpes simplex" OR herpesvirus OR simplexvirus OR "HSV-1" OR "HHV-1" OR "HSV-2" OR "HHV-2" OR syphilis OR chancre OR "treponema pallidum" OR "condylomata lata" OR chlamydia OR "lymphogranuloma venereum" OR LGV OR "lymphogranuloma inguinale" OR gonorrhea OR "neisseria gonorrhoeae" OR "gonococcal urethritis" OR gonococci OR trichomoniasis OR trichomoniases OR "trichomonas vaginalis" OR "trichomonas vaginitides" OR "trichomonas vaginitis" OR "trichomonas vaginali" OR "granuloma venereum" OR donovanosis OR chancroid$ OR "haemophilus ducreyi")

Updated search strategy Jan 2021

Syndromic management of GUD: 2021 update final search strategies

4 Table of Contents

1 Search methodology 42
2 Databases 42
2.1 Information management 43
3 Results 43
4 References 44
5 Appendix: Search strategies 44
5.1 Medline 44
5.2 Embase 46
5.3 Global Health 49
5.4 CINAHL Plus 52
5.5 Africa-Wide Information 54
5.6 Web of Science Core Collection 56
5.7 LILACS 58

5 Search methodology

The searches run in September 2019 were re-run in January 2021. No changes were made to the 2019 searches.

Full details of the 2021 search strings used for each database can be found in the appendix.

6 Databases

The following bibliographic databases were searched on 11 January 2021

- OvidSP MEDLINE ALL, 1946 to 08 January 2021.
- OvidSP Embase, 1980 to 2021 week 01.
- OvidSP Global Health, 1910 to 2021 week 01.
- Ebsco CINAHL Plus, complete database.
- Ebsco Africa-Wide Information, complete database.
- Clarivate Analytics Web of Science Core Collection, Data last updated 08 January 2021. This collection contains the following databases:
    - Science Citation Index-Expanded, 1970-present;
    - Social Sciences Citation Index, 1970-present;
    - Arts & Humanities Citation Index, 1970-present;
    - Conference Proceedings Citation Index-Science, 1990-present;

  - Conference Proceedings Citation Index-Social Science & Humanities, 1990-present;
  - Emerging Sources Citation Index, 2015-present.
- World Health Organization LILACS, complete database.

The OvidSP Northern Light database was searched in 2019. This was not available in 2021.

6.1 Information management

All citations identified by our searches were imported into EndNote X9 software. The EndNote library contained the previously retrieved and deduplicated results from the 2019 search. All results retrieved in 2021 were imported and duplicates were identified and removed using the method described on the London School of Hygiene & Tropical Medicine Library & Archives Service blog.[1] Once the 2019 results were removed, only the results retrieved by the update remained.

7 Results

Number of results pre-and post-deduplication are listed in the table below.

Database name	Total number of results	Number of results once duplicates and 2019 results removed
Medline	2008	229
Embase	5307	529
Global Health	1447	193
CINAHL Plus	540	463
Africa-Wide Information	303	0
Web of Science Core Collection databases (all searched together)	1003	50
LILACS	59	18

**Total**	**10,667**	**1482**

8 References

1. Falconer J. Removing duplicates from an EndNote Library. Library & Archives Service Blog [Internet]: London School of Hygiene & Tropical Medicine. 2018. [cited 2020]. Available from: https://blogs.lshtm.ac.uk/library/2018/12/07/removing-duplicates-from-an-endnote-library/.

9 Appendix: Search strategies

This appendix provides full details of all search strings used for bibliographic databases, with dates and number of references returned. The EndNote X9 import order is provided, as the deduplication technique keeps the first uploaded copy of the reference by default. Note that the 2019 results were uploaded first, so these results were kept by default. One the deduplication process was completed the 2019 results were removed, leaving only papers retrieved since the 2019 searches were run.

In all searches, numbers in parentheses at the end of each row show the number of hits retrieved.

9.1 Medline

Database name	Medline ALL
Database platform	OvidSP
Dates of database coverage	1946 to 08 January 2021
Date searched	11 January 2021
Searched by	JF
Number of results	2008
EndNote import order	1
Number of results once duplicates removed	229

1. ((ulcer* or vesicle* or papule* or chancr* or granulom* or sore* or lesion* or pustule?) adj3 (genital* or vener* or penile or penis or anogenital or vagin*)).ti,ab. (7031)
2. gud.ti,ab. (253)
3. Herpes Simplex/ (14446)
4. Herpes genitalis/ (4705)
5. simplexvirus/ (17288)
6. "herpesvirus 1, human"/ (10602)
7. "herpesvirus 2, human"/ (4370)
8. herpes genitalis.ti,ab. (336)
9. genital herpes.ti,ab. (3087)
10. herpes virus.ti,ab. (5650)
11. herpes simplex.ti,ab. (40831)
12. herpesvirus.ti,ab. (22776)
13. simplexvirus.ti,ab. (35)
14. "HSV-1".ti,ab. (12424)
15. "HHV-1".ti,ab. (90)
16. "HSV-2".ti,ab. (6209)
17. "HHV-2".ti,ab. (39)
18. exp Trichomonas Infections/ (6190)
19. Trichomonas vaginalis/ (3626)
20. trichomoniasis.ti,ab. (3120)
21. trichomoniases.ti,ab. (2)
22. trichomonas vaginalis.ti,ab. (4738)
23. trichomonas vaginitides.ti,ab. (0)
24. trichomonas vaginitis.ti,ab. (267)
25. trichomonas vaginali.ti,ab. (2)
26. exp Syphilis/ (27957)
27. Treponema pallidum/ (4118)
28. syphilis.ti,ab. (25959)
29. chancre.ti,ab. (507)
30. treponema pallidum.ti,ab. (4331)
31. condylomata lata.ti,ab. (47)
32. Granuloma Inguinale/ (592)
33. Calymmatobacterium/ (71)
34. granuloma inguinale.ti,ab. (358)
35. Calymmatobacterium granulomatis.ti,ab. (37)
36. granuloma venereum.ti,ab. (67)
37. donovanosis.ti,ab. (237)
38. Chlamydia/ (2976)
39. chlamydia trachomatis/ (12153)
40. chlamydia.ti,ab. (25327)
41. lymphogranuloma venereum/ (1601)
42. lymphogranuloma venereum.ti,ab. (1129)
43. LGV.ti,ab. (589)

44. lymphogranuloma inguinale.ti,ab. (84)
45. Chancroid/ (864)
46. Haemophilus ducreyi/ (656)
47. chancroid?.ti,ab. (893)
48. haemophilus ducreyi.ti,ab. (767)
49. hemophilus ducreyi.ti,ab. (37)
50. Gonorrhea/ (14097)
51. Neisseria gonorrhoeae/ (9966)
52. gonorrhea.ti,ab. (7194)
53. neisseria gonorrhoeae.ti,ab. (9906)
54. gonococcal urethritis.ti,ab. (1273)
55. gonococci.ti,ab. (2036)
56. or/1-55 [GENITAL ULCER DISEASE] (169172)
57. Syndrome/ (116841)
58. algorithms/ (261377)
59. Practice Guidelines as Topic/ (121541)
60. Risk Assessment/ (274543)
61. Critical Pathways/ (7004)
62. flowchart?.ti,ab. (1389)
63. flow-chart?.ti,ab. (1544)
64. algorithm?.ti,ab. (262860)
65. clinical pathway?.ti,ab. (3644)
66. risk assessment?.ti,ab. (68326)
67. syndromically.ti,ab. (47)
68. syndromic.ti,ab. (12326)
69. decision tree.ti,ab. (7887)
70. detection tool?.ti,ab. (1327)
71. or/57-70 [SYNDROMIC MANAGEMENT] (968921)
72. exp "Sensitivity and Specificity"/ (596418)
73. effective*.ti,ab. (2049440)
74. sensitiv*.ti,ab. (1429401)
75. specific*.ti,ab. (3223083)
76. perfor*.ti,ab. (3488771)
77. predict*.ti,ab. (1652705)
78. quantitative.ti,ab. (638900)
79. random*.ti,ab. (1190416)
80. predictive value?.ti,ab. (112958)
81. study.ab. (7389049)
82. efficacy.ti,ab. (840955)
83. (accuracy or accurate).ti,ab. (720548)
84. or/72-83 [SENSITIVITY AND SPECIFICITY] (13931817)
85. 56 and 71 (3347)
86. 84 and 85 (2008)

9.2 Embase

MONASH University

Database name	Embase
Database platform	OvidSP
Dates of database coverage	1980 to 2021 week 01
Date searched	11 January 2021
Searched by	JF
Number of results	5307
EndNote import order	2
Number of results once duplicates removed	529

1. syndrome/ (43365)
2. algorithm/ (284385)
3. practice guideline/ (444233)
4. risk assessment/ (594236)
5. clinical pathway/ (8657)
6. "decision tree"/ (14009)
7. flowchart?.ti,ab. (2166)
8. flow-chart?.ti,ab. (2485)
9. algorithm?.ti,ab. (336090)
10. clinical pathway?.ti,ab. (5983)
11. risk assessment?.ti,ab. (90640)
12. syndromically.ti,ab. (73)
13. syndromic.ti,ab. (16718)
14. decision tree.ti,ab. (11814)
15. detection tool?.ti,ab. (1682)
16. or/1-15 [SYNDROMIC MANAGEMENT] (1526993)
17. "sensitivity and specificity"/ (379831)
18. clinical effectiveness/ (135329)
19. predictive value/ (182723)
20. diagnostic accuracy/ (260158)

21. effective*.ti,ab. (2647712)
22. sensitiv*.ti,ab. (1750102)
23. specific*.ti,ab. (3977280)
24. perfor*.ti,ab. (4819423)
25. predict*.ti,ab. (2213151)
26. quantitative.ti,ab. (771387)
27. random*.ti,ab. (1609741)
28. predictive value?.ti,ab. (168740)
29. study.ab. (9835919)
30. efficacy.ti,ab. (1226401)
31. (accuracy or accurate).ti,ab. (909642)
32. or/17-31 [SENSITIVITY AND SPECIFICITY] (17628198)
33. genital ulcer/ (4044)
34. ((ulcer* or vesicle* or papule* or chancr* or granulom* or sore* or lesion* or pustule?) adj3 (genital* or vener* or penile or penis or anogenital or vagin*)).ti,ab. (9223)
35. gud.ti,ab. (291)
36. herpes simplex/ (17834)
37. genital herpes/ (5878)
38. herpes labialis/ (2057)
39. simplexvirus/ (960)
40. exp Herpes simplex virus/ (32496)
41. herpes genitalis.ti,ab. (371)
42. genital herpes.ti,ab. (3654)
43. herpes virus.ti,ab. (7157)
44. herpes simplex.ti,ab. (43875)
45. herpesvirus.ti,ab. (23211)
46. simplexvirus.ti,ab. (47)
47. "HSV-1".ti,ab. (14447)
48. "HHV-1".ti,ab. (113)
49. "HSV-2".ti,ab. (7137)
50. "HHV-2".ti,ab. (54)
51. exp Trichomonas/ (6767)
52. trichomoniasis.ti,ab. (2645)
53. trichomoniases.ti,ab. (1)
54. trichomonas vaginalis.ti,ab. (4896)
55. trichomonas vaginitides.ti,ab. (0)
56. trichomonas vaginitis.ti,ab. (98)
57. trichomonas vaginali.ti,ab. (0)
58. exp syphilis/ (23348)
59. Treponema pallidum/ (5739)
60. syphilis.ti,ab. (22226)
61. chancre.ti,ab. (383)
62. treponema pallidum.ti,ab. (4487)
63. condylomata lata.ti,ab. (55)
64. granuloma inguinale/ (532)
65. klebsiella/ (14745)
66. klebsiella granulomatis/ (25)

67. granuloma inguinale.ti,ab. (184)
68. Calymmatobacterium granulomatis.ti,ab. (35)
69. granuloma venereum.ti,ab. (14)
70. donovanosis.ti,ab. (249)
71. chlamydia/ (7455)
72. chlamydia trachomatis/ (18877)
73. chlamydia.ti,ab. (31711)
74. lymphogranuloma venereum/ (1392)
75. lymphogranuloma venereum.ti,ab. (1017)
76. LGV.ti,ab. (815)
77. lymphogranuloma inguinale.ti,ab. (11)
78. ulcus molle/ (1160)
79. haemophilus ducreyi/ (1048)
80. chancroid?.ti,ab. (817)
81. haemophilus ducreyi.ti,ab. (828)
82. hemophilus ducreyi.ti,ab. (16)
83. gonorrhea/ (15190)
84. gonococcal urethritis/ (675)
85. Neisseria gonorrhoeae/ (14588)
86. gonorrhea.ti,ab. (6405)
87. neisseria gonorrhoeae.ti,ab. (10765)
88. gonococcal urethritis.ti,ab. (1226)
89. gonococci.ti,ab. (1686)
90. or/33-89 [GENITAL ULCER SYNDROME] (198383)
91. 16 and 90 (8270)
92. 32 and 91 (5352)
93. remove duplicates from 92 (5307)

9.3 Global Health

Database name	Global Health
Database platform	OvidSP
Dates of database coverage	1910 to week 01 2021
Date searched	11 January 2021
Searched by	JF

Number of results	1447
EndNote import order	3
Number of results once duplicates removed	193

1. syndromic management/ (17)
2. algorithms/ (6410)
3. guidelines/ (53775)
4. risk assessment/ (63423)
5. flowchart?.ti,ab. (214)
6. flow-chart?.ti,ab. (319)
7. algorithm?.ti,ab. (15993)
8. clinical pathway?.ti,ab. (295)
9. risk assessment?.ti,ab. (25197)
10. syndromically.ti,ab. (23)
11. syndromic.ti,ab. (1920)
12. decision tree.ti,ab. (1307)
13. detection tool?.ti,ab. (246)
14. or/1-13 [SYNDROMIC MANAGEMENT] (139789)
15. sensitivity analysis/ (1700)
16. program effectiveness/ (119)
17. diagnostic value/ (4859)
18. effective*.ti,ab. (385230)
19. sensitiv*.ti,ab. (230090)
20. specific*.ti,ab. (457336)
21. perfor*.ti,ab. (443948)
22. predict*.ti,ab. (200411)
23. quantitative.ti,ab. (94837)
24. random*.ti,ab. (236078)
25. predictive value?.ti,ab. (16944)
26. study.ab. (1484235)
27. efficacy.ti,ab. (137175)
28. (accuracy or accurate).ti,ab. (83071)
29. or/15-28 [SENSITIVITY AND SPECIFICITY] (2349049)
30. genital ulcers/ (722)
31. ((ulcer* or vesicle* or papule* or chancr* or granulom* or sore* or lesion* or pustule?) adj3 (genital* or vener*or penile or penis or anogenital or vagin*)).ti,ab. (2278)
32. gud.ti,ab. (144)
33. exp neisseria gonorrhoeae/ (10634)
34. gonorrhea.ti,ab. (2491)
35. neisseria gonorrhoeae.ti,ab. (4846)
36. gonococcal urethritis.ti,ab. (962)

37. gonococci.ti,ab. (1673)
38. chlamydia/ (12570)
39. chlamydia trachomatis/ (9688)
40. chlamydia.ti,ab. (11643)
41. lymphogranuloma venereum.ti,ab. (862)
42. LGV.ti,ab. (313)
43. lymphogranuloma inguinale.ti,ab. (375)
44. exp herpes simplex/ (7141)
45. simplexvirus/ (5582)
46. human herpesvirus 1/ (2991)
47. human herpesvirus 2/ (2981)
48. herpes genitalis.ti,ab. (153)
49. genital herpes.ti,ab. (1452)
50. herpes virus.ti,ab. (1858)
51. herpes simplex.ti,ab. (10444)
52. herpesvirus.ti,ab. (5170)
53. simplexvirus.ti,ab. (13)
54. "HSV-1".ti,ab. (2659)
55. "HHV-1".ti,ab. (49)
56. "HSV-2".ti,ab. (2606)
57. "HHV-2".ti,ab. (21)
58. exp treponema pallidum/ (18106)
59. syphilis.ti,ab. (17434)
60. chancre.ti,ab. (531)
61. treponema pallidum.ti,ab. (2757)
62. condylomata lata.ti,ab. (15)
63. Trichomonas/ (7030)
64. Trichomonas vaginalis/ (5139)
65. trichomoniasis.ti,ab. (2340)
66. trichomoniases.ti,ab. (2)
67. trichomonas vaginalis.ti,ab. (4755)
68. trichomonas vaginitides.ti,ab. (0)
69. trichomonas vaginitis.ti,ab. (132)
70. trichomonas vaginali.ti,ab. (0)
71. exp klebsiella granulomatis/ (572)
72. granuloma inguinale.ti,ab. (429)
73. Calymmatobacterium granulomatis.ti,ab. (27)
74. granuloma venereum.ti,ab. (180)
75. donovanosis.ti,ab. (122)
76. exp haemophilus ducreyi/ (823)
77. chancroid?.ti,ab. (692)
78. haemophilus ducreyi.ti,ab. (336)
79. hemophilus ducreyi.ti,ab. (11)
80. or/30-79 [GENITAL ULCER DISEASE] (64356)

81. 14 and 80 (1978)
82. 29 and 81 (1447)

9.4 CINAHL Plus

Database name	CINAHL Plus
Database platform	Ebsco
Dates of database coverage	Complete database to search date
Date searched	11 January 2021
Searched by	JF
Number of results	540
EndNote import order	4
Number of results once duplicates removed	463

S1 (TI (ulcer* or vesicle* or papule* or chancr* or granulom* or sore* or lesion* or pustule#) N3 (genital* or vener* or penile or penis or anogenital or vagin*)) OR (AB (ulcer* or vesicle* or papule* or chancr* or granulom* or sore* or lesion* or pustule#) N3 (genital* or vener* or penile or penis or anogenital or vagin*)) (1,342)
S2 (TI gud) OR (AB gud) (66)
S3 (MH "Herpes Simplex") (2,385)
S4 (MH "Herpes Genitalis") (1,424)
S5 (MH "Herpesviruses") (3,624)
S6 (TI "herpes genitalis") OR (AB "herpes genitalis") (31)
S7 (TI "genital herpes") OR (AB "genital herpes") (768)
S8 (TI "herpes virus") OR (AB "herpes virus") (435)
S9 (TI "herpes simplex") OR (AB "herpes simplex") (3,573)
S10 (TI herpesvirus) OR (AB herpesvirus) (1,093)
S11 (TI simplexvirus) OR (AB simplexvirus) (1)
S12 (TI "HSV-1") OR (AB "HSV-1") (562)
S13 (TI "HHV-1") OR (AB "HHV-1") (6)
S14 (TI "HSV-2") OR (AB "HSV-2") (799)
S15 (TI "HHV-2") OR (AB "HHV-2") (2)

S16 (MH "Syphilis+") (4,181)
S17 (TI syphilis) OR (AB syphilis) (4,508)
S18 (TI chancre) OR (AB chancre) (47)
S19 (TI "treponema pallidum") OR (AB "treponema pallidum") (552)
S20 (TI "condylomata lata") OR (AB "condylomata lata") (4)
S21 (MH "Chlamydia Infections") (4,329)
S22 (MH "Lymphogranuloma Venereum") (229)
S23 (MH "Chlamydia+") (2,288)
S24 (TI chlamydia) OR (AB chlamydia) (5,280)
S25 (TI "lymphogranuloma venereum") OR (AB "lymphogranuloma venereum") (188)
S26 (TI LGV) OR (AB LGV) (87)
S27 (TI "lymphogranuloma inguinale") OR (AB "lymphogranuloma inguinale") (0)
S28 (MH "Gonorrhea") (3,227)
S29 (MH "Neisseria") (1,387)
S30 (TI gonorrhea) OR (AB gonorrhea) (2,517)
S31 (TI "neisseria gonorrhoeae") OR (AB "neisseria gonorrhoeae") (1,563)
S32 (TI "gonococcal urethritis") OR (AB "gonococcal urethritis") (110)
S33 (TI gonococci) OR (AB gonococci) (110)
S34 (MH "Trichomonas Vaginitis") (541)
S35 (TI trichomoniasis) OR (AB trichomoniasis) (506)
S36 (TI trichomoniases) OR (AB trichomoniases) (1)
S37 (TI "trichomonas vaginalis") OR (AB "trichomonas vaginalis") (726)
S38 (TI "trichomonas vaginali") OR (AB "trichomonas vaginali") (0)
S39 (MH "Granuloma Inguinale") (32)
S40 (MH "Klebsiella") (1,730)
S41 (TI "granuloma inguinale") OR (AB "granuloma inguinale") (13)
S42 (TI "Calymmatobacterium granulomatis") OR (AB "Calymmatobacterium granulomatis") (6)
S43 (TI "granuloma venereum") OR (AB "granuloma venereum") (0)
S44 (TI donovanosis) OR (AB donovanosis) (24)
S45 (MH "Chancroid") (46)
S46 (TI chancroid*) OR (AB chancroid*) (91)
S47 (TI "haemophilus ducreyi") OR (AB "haemophilus ducreyi") (77)
S48 (TI "hemophilus ducreyi") OR (AB "hemophilus ducreyi") (2)
S49 S1 or S2 or S3 or S4 or S5 or S6 or S7 or S8 or S9 or S10 or S11 or S12 or S13 or S14 or S15 or S16 or S17 or S18 or S19 or S20 or S21 or S22 or S23 or S24 or S25 or S26 or S27 or S28 or S29 or S30 or S31 or S32 or S33 or S34 or S35 or S36 or S37 or S38 or S39 or S40 or S41 or S42 or S43 or S44 or S45 or S46 or S47 or S48 (25,017)
S50 (MH "Syndrome") (12,024)
S51 (MH "Algorithms") (38,540)
S52 (MH "Decision Trees") (2,474)
S53 (MH "Practice Guidelines") (80,004)
S54 (MH "Risk Assessment") (110,687)
S55 (MH "Critical Path") (5,463)
S56 (TI flowchart#) OR (AB flowchart#) (536)

S57 (TI "flow-chart#") OR (AB "flow-chart#") (412)
S58 (TI algorithm#) OR (AB algorithm#) (35,933)
S59 (TI "clinical pathway#") OR (AB "clinical pathway#") (2,004)
S60 (TI "risk assessment#") OR (AB "risk assessment#") (16,824)
S61 (TI syndromically) OR (AB syndromically) (15)
S62 (TI syndromic) OR (AB syndromic) (2,401)
S63 (TI "decision tree") OR (AB "decision tree") (1,987)
S64 (TI "detection tool#") OR (AB "detection tool#") (203)
S65 S50 OR S51 OR S52 OR S53 OR S54 OR S55 OR S56 OR S57 OR S58 OR S59 OR S60 OR S61 OR S62 OR S63 OR S64 (271,087)
S66 (MH "Sensitivity and Specificity") (85,439)
S67 (MH "Predictive Value of Tests") (52,286)
S68 (TI effective*) OR (AB effective*) (466,305)
S69 (TI sensitiv*) OR (AB sensitiv*) (166,313)
S70 (TI specific*) OR (AB specific*) (423,998)
S71 (TI perfor*) or (AB perfor*) (626,715)
S72 (TI predict*) OR (AB predict*) (370,887)
S73 (TI quantitative) OR (AB quantitative) (86,996)
S74 (TI random*) OR (AB random*) (363,686)
S75 (TI "predictive value#") OR (AB "predictive value#") (27,413)
S76 (AB study) (1,870,391)
S77 (TI efficacy) OR (AB efficacy) (197,083)
S78 (TI (accuracy or accurate)) OR (AB (accuracy or accurate)) (123,766)
S79 S66 OR S67 OR S68 OR S69 OR S70 OR S71 OR S72 OR S73 OR S74 OR S75 OR S76 OR S77 OR S78 (2,713,404)
S80 S49 AND S65 (1,245)
S81 S79 AND S80 (693)
S82 S80 NOT S81 (552)
S83 S81 Published Date: 19950101-20201231 (686)
S84 S82 Published Date: 19950101-20201231 (540)

9.5 Africa-Wide Information

Database name	Africa-Wide Information
Database platform	Ebsco
Dates of database coverage	Complete database to search date
Date searched	11 January 2021
Searched by	JF

Number of results	303
EndNote import order	5
Number of results once duplicates removed	0

S1 (TI (ulcer* or vesicle* or papule* or chancr* or granulom* or sore* or lesion* or pustule#) N3 (genital* or vener* or penile or penis or anogenital or vagin*)) OR (AB (ulcer* or vesicle* or papule* or chancr* or granulom* or sore* or lesion* or pustule#) N3 (genital* or vener* or penile or penis or anogenital or vagin*)) (1,326)
S2 (TI gud) OR (AB gud) (141)
S3 (TI "herpes genitalis") OR (AB "herpes genitalis") (29)
S4 (TI "genital herpes") OR (AB "genital herpes") (296)
S5 (TI "herpes virus") OR (AB "herpes virus") (391)
S6 (TI "herpes simplex") OR (AB "herpes simplex") (1,954)
S7 (TI herpesvirus) OR (AB herpesvirus) (1,290)
S8 (TI simplexvirus) OR (AB simplexvirus) (1)
S9 (TI "HSV-1") OR (AB "HSV-1") (371)
S10 (TI "HHV-1") OR (AB "HHV-1") (5)
S11 (TI "HSV-2") OR (AB "HSV-2") (648)
S12 (TI "HHV-2") OR (AB "HHV-2") (4)
S13 (TI syphilis) OR (AB syphilis) (3,372)
S14 (TI chancre) OR (AB chancre) (114)
S15 (TI "treponema pallidum") OR (AB "treponema pallidum") (482)
S16 (TI "condylomata lata") OR (AB "condylomata lata") (2)
S17 (TI chlamydia) OR (AB chlamydia) (2,176)
S18 (TI "lymphogranuloma venereum") OR (AB "lymphogranuloma venereum") (116)
S19 (TI LGV) OR (AB LGV) (48)
S20 (TI "lymphogranuloma inguinale") OR (AB "lymphogranuloma inguinale") (3)
S21 (TI gonorrhea) OR (AB gonorrhea) (848)
S22 (TI "neisseria gonorrhoeae") OR (AB "neisseria gonorrhoeae") (990)
S23 (TI "gonococcal urethritis") OR (AB "gonococcal urethritis") (108)
S24 (TI gonococci) OR (AB gonococci) (109)
S25 (TI trichomoniasis) OR (AB trichomoniasis) (465)
S26 (TI trichomoniases) OR (AB trichomoniases) (3)
S27 (TI "trichomonas vaginalis") OR (AB "trichomonas vaginalis") (677)
S28 (TI "trichomonas vaginali") OR (AB "trichomonas vaginali") (0)
S29 (TI "granuloma inguinale") OR (AB "granuloma inguinale") (67)
S30 (TI "Calymmatobacterium granulomatis") OR (AB "Calymmatobacterium granulomatis") (17)
S31 (TI "granuloma venereum") OR (AB "granuloma venereum") (4)

S32 (TI donovanosis) OR (AB donovanosis) (57)
S33 (TI chancroid*) OR (AB chancroid*) (271)
S34 (TI "haemophilus ducreyi") OR (AB "haemophilus ducreyi") (178)
S35 (TI "hemophilus ducreyi") OR (AB "hemophilus ducreyi") (11)
S36 S1 or S2 or S3 or S4 or S5 or S6 or S7 or S8 or S9 or S10 or S11 or S12 or S13 or S14 or S15 or S16 or S17 or S18 or S19 or S20 or S21 or S22 or S23 or S24 or S25 or S26 or S27 or S28 or S29 or S30 or S31 or S32 or S33 or S34 or S35 (11,291)
S37 (TI flowchart#) OR (AB flowchart#) (114)
S38 (TI "flow-chart#") OR (AB "flow-chart#") (263)
S39 (TI algorithm#) OR (AB algorithm#) (8,342)
S40 (TI "clinical pathway#") OR (AB "clinical pathway#") (87)
S41 (TI "risk assessment#") OR (AB "risk assessment#") (3,497)
S42 (TI syndromically) OR (AB syndromically) (19)
S43 (TI syndromic) OR (AB syndromic) (916)
S44 (TI "decision tree") OR (AB "decision tree") (330)
S45 (TI "detection tool#") OR (AB "detection tool#") (74)
S46 S37 OR S38 OR S39 OR S40 OR S41 OR S42 OR S43 OR S44 OR S45 (13,373)
S47 (TI effective*) OR (AB effective*) (140,925)
S48 (TI sensitiv*) OR (AB sensitiv*) (65,052)
S49 (TI specific*) OR (AB specific*) (176,524)
S50 (TI perfor*) or (AB perfor*) (172,945)
S51 (TI predict*) OR (AB predict*) (77,568)
S52 (TI quantitative) OR (AB quantitative) (28,098)
S53 (TI random*) OR (AB random*) (64,888)
S54 (TI "predictive value#") OR (AB "predictive value#") (5,684)
S55 (AB study) (465,864)
S56 (TI efficacy) OR (AB efficacy) (40,619)
S57 (TI (accuracy or accurate)) OR (AB (accuracy or accurate)) (31,312)
S58 S47 OR S48 OR S49 OR S50 OR S51 OR S52 OR S53 OR S54 OR S55 OR S56 OR S57 (831,602)
S59 S36 AND S46 (372)
S60 S58 AND S59 (303)

9.6 Web of Science Core Collection

Database name	Web of Science Core Collection. This contains the following databases which are all searched together:<br><br>• Science Citation Index Expanded (SCI-Expanded)<br>• Social Sciences Citation Index (SSCI)<br>• Arts & Humanities Citation Index (A&HCI)<br>• Conference Proceedings Citation Index-Science (CPCI-S)<br>• Conference Proceedings Citation Index-Social Science & Humanities (CPCI-SSH)

	• Emerging Sources Citation Index (ESCI)
Database platform	Clarivate Analytics Web of Science
Dates of database coverage	SCI-Expanded, 1970-present<br>SSCI, 1970-present<br>A&HCI, 1970-present<br>CPCI-S, 1990-present<br>CPCI-SSH, 1990-present<br>ESCI, 2015-present<br><br>Data last updated: 2021-01-08
Date searched	11 January 2021
Searched by	JF
Number of results	1003
EndNote import order	6
Number of results once duplicates removed	50

All searches run across the following options: Indexes=SCI-EXPANDED, SSCI, A&HCI, CPCI-S, CPCI-SSH, ESCI Timespan=All years

#1 TOPIC: (flowchart$ or flow-chart$ or algorithm$ or "clinical pathway$" or "risk assessment$" or syndromically or syndromic or "decision tree" or "detection tool$") (2,247,334)

#2 TOPIC: (effective* or sensitiv* or specific* or perfor* or predict* or quantitative or random* or "predictive value$" or efficacy or accuracy or accurate) (20,101,625)

#3 TOPIC: (gud or "herpes genitalis" or "genital herpes" or "herpes virus" or "herpes simplex" or herpesvirus or simplexvirus or "hsv-1" or "hhv-1" or "hsv-2" or "hhv-2" or syphilis or chancre or "treponema pallidum" or "condylomata lata" or chlamydia or "lymphogranuloma venereum" or lgv or "lymphogranuloma inguinale" or gonorrhea or "neisseria gonorrhoea" or "gonococcal urethritis" or gonococci or trichomoniasis or trichomoniases or "trichomonas vaginalis" or "trichomonas vaginitides" or "trichomonas vaginitis" or "trichomonas vaginali" or "granuloma

inguinale" or "calymmatobacterium granulomatis" or "granuloma venereum" or donovanosis or chancroid$ or "haemophilus ducreyi" or "hemophilus ducreyi") (136,064)

#4 TOPIC: (((ulcer* or vesicle* or papule* or chancr* or granulom* or sore* or lesion* or pustule$) NEAR/3 genital*) or ((ulcer* or vesicle* or papule* or chancr* or granulom* or sore* or lesion* or pustule$) NEAR/3 vener*) or ((ulcer* or vesicle* or papule* or chancr* or granulom* or sore* or lesion* or pustule$) NEAR/3 penile) or ((ulcer* or vesicle* or papule* or chancr* or granulom* or sore* or lesion* or pustule$) NEAR/3 penis) or ((ulcer* or vesicle* or papule* or chancr* or granulom* or sore* or lesion* or pustule$) NEAR/3 anogenital) or ((ulcer* or vesicle* or papule* or chancr* or granulom* or sore* or lesion* or pustule$) NEAR/3 vagin*)) (6,375)

#5 #4 OR #3 (140,689)

#6 #5 AND #1 (1,414)

#7 #6 AND #2 (1,003)

9.7 LILACS

Database name	LILACS
Database platform	World Health Organization
Dates of database coverage	Complete database
Date searched	11 January 2021
Searched by	JF
Number of results	59
EndNote import order	7
Number of results once duplicates removed	18

flowchart* OR "flow-chart*" OR algorithm* OR "clinical pathway*" OR "risk assessment*" OR syndromically OR syndromic OR "decision tree" OR "detection tool*" AND effective* OR sensitiv* OR specific* OR perfor* OR predict* OR quantitative OR random* OR "predictive value*" OR efficacy OR accuracy OR accurate AND ((ulcer* OR vesicle* or papule* OR chancr* OR granulom* OR sore* OR lesion* OR pustule*) AND (genital* OR vener* OR penile OR penis OR anogenital OR vagin*)) OR "herpes genitalis" OR "herpes virus" OR "herpes simplex" OR herpesvirus OR simplexvirus OR "HSV-1" OR "HHV-1" OR "HSV-2" OR "HHV-2" OR syphilis OR chancre OR

"treponema pallidum" OR "condylomata lata" OR chlamydia OR "lymphogranuloma venereum" OR LGV OR "lymphogranuloma inguinale" OR gonorrhea OR "neisseria gonorrhoeae" OR "gonococcal urethritis" OR gonococci OR trichomoniasis OR trichomoniases OR "trichomonas vaginalis" OR "trichomonas vaginitides" OR "trichomonas vaginitis" OR "trichomonas vaginali" OR "granuloma venereum" OR donovanosis OR chancroid* OR "haemophilus ducreyi"

REFERENCES

Bibliography

1. Sanchez J, Volquez C, Totten PA, et al. The etiology and management of genital ulcers in the Dominican Republic and Peru. *Sexually Transmitted Diseases* 2002; **29**(10): 559-67.
2. Behets FM, Andriamiadana J, Randrianasolo D, et al. Chancroid, primary syphilis, genital herpes, and lymphogranuloma venereum in Antananarivo, Madagascar. *Journal of Infectious Diseases* 1999; **180**(4): 1382-5.
3. Behets FM, Brathwaite AR, Hylton-Kong T, et al. Genital ulcers: etiology, clinical diagnosis, and associated human immunodeficiency virus infection in Kingston, Jamaica. *Clinical Infectious Diseases* 1999; **28**(5): 1086-90.
4. Beyrer C, Jitwatcharanan K, Natpratan C, et al. Molecular methods for the diagnosis of genital ulcer disease in a sexually transmitted disease clinic population in northern Thailand: predominance of herpes simplex virus infection. *Journal of Infectious Diseases* 1998; **178**(1): 243-6.
5. Bhavsar C, Patel RM, Marfatia Y. A study of 113 cases of genital ulcerative disease and urethral discharge syndrome with validation of syndromic management of sexually transmitted diseases. *Indian Journal Of Sexually Transmitted Diseases And AIDS* 2014; **35**(1): 35-9.
6. Bogaerts J, Vuylsteke B, Martinez Tello W, et al. Simple algorithms for the management of genital ulcers: evaluation in a primary health care centre in Kigali, Rwanda. *Bulletin of the World Health Organization* 1995; **73**(6): 761-7.
7. DiCarlo RP, Martin DH. The clinical diagnosis of genital ulcer disease in men. *Clin Infect Dis* 1997; **25**(2): 292-8.
8. Hina R, Tankhiwale SS, Surpam RB. Study of seroprevalence and syndromic validation of HSV2 among genito-ulcerative disease patients attending STI clinic in a tertiary care hospital. *Journal of Evolution of Medical and Dental Sciences* 2017; **6**(36): 2984-6.
9. Htun Y, Morse SA, Dangor Y, et al. Comparison of clinically directed, disease specific, and syndromic protocols for the management of genital ulcer disease in Lesotho. *Sexually Transmitted Infections* 1998; **74 Suppl 1**: S23-8.
10. Prabhakar P, Narayanan P, Deshpande GR, et al. Genital ulcer disease in India: etiologies and performance of current syndrome guidelines. *Sexually Transmitted Diseases* 2012; **39**(11): 906-10.
11. Risbud A, Chan-Tack K, Gadkari D, et al. The etiology of genital ulcer disease by multiplex polymerase chain reaction and relationship to HIV infection among patients attending sexually transmitted disease clinics in Pune, India. *Sex Transm Dis* 1999; **26**(1): 55-62.
12. Wang Q, Yang P, Zhong M, Wang G. Validation of diagnostic algorithms for syndromic management of sexually transmitted diseases. *Chinese Medical Journal* 2003; **116**(2): 181-6.
13. Wang QQ, Mabey D, Peeling RW, et al. Validation of syndromic algorithm for the management of genital ulcer diseases in China. *International Journal of STD & AIDS* 2002; **13**(7): 469-74.
14. Fast MV, D'Costa LJ, Nsanze H, et al. The clinical diagnosis of genital ulcer disease in men in the tropics. *Sex Transm Dis* 1984; **11**(2): 72-6.
15. Dangor Y, Ballard RC, da LEF, Fehler G, Miller SD, Koornhof HJ. Accuracy of clinical diagnosis of genital ulcer disease. *Sex Transm Dis* 1990; **17**(4): 184-9.
16. Hanson S, Sunkutu RM, Kamanga J, Hojer B, Sandstrom E. STD care in Zambia: an evaluation of the guidelines for case management through a syndromic approach. *International Journal of STD & AIDS* 1996; **7**(5): 324-32.

17. Ndinya-Achola JO, Kihara AN, Fisher LD, et al. Presumptive specific clinical diagnosis of genital ulcer disease (GUD) in a primary health care setting in Nairobi. *International Journal of STD & AIDS* 1996; **7**(3): 201-5.
18. Choudhry S, Ramachandran VG, Das S, Bhattacharya SN, Mogha NS. Pattern of sexually transmitted infections and performance of syndromic management against etiological diagnosis in patients attending the sexually transmitted infection clinic of a tertiary care hospital. *Indian Journal Of Sexually Transmitted Diseases And AIDS* 2010; **31**(2): 104-8.
19. Clark JL, Lescano AG, Konda KA, et al. Syndromic management and STI control in urban Peru. *PLoS One* 2009; **4**(9): e7201.
20. Daly CC, Maggwa N, Mati JK, et al. Risk factors for gonorrhoea, syphilis, and trichomonas infections among women attending family planning clinics in Nairobi, Kenya. *Genitourinary Medicine* 1994; **70**(3): 155-61.
21. Das A, Ghosh P, Ghosh I, et al. Usefulness and Utility of NACO Regime in the Management of Sexually Transmitted Infections: A Pilot Study. *Indian Journal of Dermatology* 2017; **62**(6): 630-4.
22. Desai VK, Kosambiya JK, Thakor HG, Umrigar DD, Khandwala BR, Bhuyan KK. Prevalence of sexually transmitted infections and performance of STI syndromes against aetiological diagnosis, in female sex workers of red light area in Surat, India. *Sexually Transmitted Infections* 2003; **79**(2): 111-5.
23. Liu H, Jamison D, Li X, Ma E, Yin Y, Detels R. Is syndromic management better than the current approach for treatment of STDs in China? Evaluation of the cost-effectiveness of syndromic management for male STD patients. *Sexually Transmitted Diseases* 2003; **30**(4): 327-30.
24. Muralidhar S, Talwar R, Anil Kumar D, et al. Genital Ulcer Disease: How Worrisome Is It Today? A Status Report from New Delhi, India. *Journal of Sexually Transmitted Diseases Print* 2013; **2013**: 203636.
25. O'Farrell N, Morison L, Moodley P, et al. High-risk sexual behaviour in men attending a sexually transmitted infection clinic in Durban, South Africa. *Sexually Transmitted Infections* 2007; **83**(7): 530-3.
26. Otieno FO, Ndivo R, Oswago S, et al. Evaluation of syndromic management of sexually transmitted infections within the Kisumu Incidence Cohort Study. *International Journal of STD & AIDS* 2014; **25**(12): 851-9.
27. Shah NS, Kim E, de Maria Hernandez Ayala F, et al. Performance and comparison of self-reported STI symptoms among high-risk populations - MSM, sex workers, persons living with HIV/AIDS - in El Salvador. *International Journal of STD & AIDS* 2014; **25**(14): 984-91.
28. Shahesmaeili A, Karamouzian M, Shokoohi M, et al. Symptom-Based Versus Laboratory-Based Diagnosis of Five Sexually Transmitted Infections in Female Sex Workers in Iran. *Aids and Behavior* 2018; **22**: S19-S25.
29. Tsai CH, Lee TC, Chang HL, Tang LH, Chiang CC, Chen KT. The cost-effectiveness of syndromic management for male sexually transmitted disease patients with urethral discharge symptoms and genital ulcer disease in Taiwan. *Sexually Transmitted Infections* 2008; **84**(5): 400-4.
30. Yu MC, Li LH, Lu TH, Tang LH, Tsai CH, Chen KT. Aetiology of sexually transmitted disease (STD) and comparison of STD syndromes and aetiological diagnosis in Taipei, Taiwan. *Clinical Microbiology & Infection* 2005; **11**(11): 914-8.
